# A strategy for liver selective NRF2 induction *via* cytochrome P450-activated prodrugs with low activity in hypoxia

**DOI:** 10.1016/j.jbc.2025.108487

**Published:** 2025-04-08

**Authors:** Mei Ying Ng, Thilo Hagen

**Affiliations:** Department of Biochemistry, Yong Loo Lin School of Medicine, National University of Singapore, Singapore

**Keywords:** NRF2, KEAP1, prodrugs, hypoxia, cytochrome P450, NASH

## Abstract

Activation of the transcription factor nuclear factor-erythroid 2-related factor 2 (NRF2) has been shown to be a promising therapeutic approach in the treatment of hepatosteatosis. NRF2 is believed to exert beneficial effects by upregulating cellular oxidative defense mechanisms and inhibiting inflammation. However, a major concern associated with long-term treatment with NRF2 activators are drug side effects, including the promotion of tumorigenesis. Many NRF2 activators function by forming cysteine adducts with KEAP1, which normally mediates the ubiquitination and degradation of NRF2. In this study, we identified NRF2 activator prodrugs of 4-methylcatechol and tert-butylhydroquinone. These prodrugs are converted into their active metabolites in a liver selective, cytochrome P450-dependent manner and function by inhibiting KEAP1, resulting in NRF2 activation. Unexpectedly, we also found that a number of NRF2-activating compounds, including 4-methylcatechol and tert-butylhydroquinone, show a markedly lower activity under hypoxic conditions than normoxia. Our findings suggest that the lower activity of these NRF2 inducers is a consequence of less potent cysteine adduct formation with KEAP1. The lower activity of NRF2 inducing compounds in hypoxia may limit tumor promoting effects of NRF2 induction. Our study provides an important proof of concept that it is possible to selectively activate NRF2 in the liver for the treatment of hepatosteatosis while avoiding tumorigenic effects as well as side effects of NRF2 activation in other tissues.

Nonalcoholic fatty liver disease (NAFLD) and nonalcoholic steatohepatitis (NASH) are of major concern worldwide due to their increasing prevalence and because they are important risk factors for type 2 diabetes and cardiovascular disease (1, 2). In addition, NAFLD and NASH frequently progress to liver cirrhosis and hepatocellular carcinoma, and liver disease is a major cause of death in individuals with NAFLD and NASH (3). Hence, there is an urgent need to develop new therapeutics for these conditions.

One very promising approach for the treatment of NAFLD and NASH that has recently emerged is activation of the transcription factor nuclear factor-erythroid 2-related factor 2 (NRF2). Thus, deletion of NRF2 was found to result in rapid progression of steatohepatitis in high-fat diet fed mice (4, 5), while activation of NRF2 with sulforaphane led to reduced hepatic glucose production and improved glucose control in patients with type 2 diabetes, one of the major metabolic diseases associated with NASH (6). NRF2 is known as a master regulator of the cellular phase 2 response to inactivate drugs and xenobiotics. In addition, NRF2 plays an important role in the cellular antioxidant defense by upregulating the expression of various reactive oxygen species scavenging and other antioxidant enzymes as well as in mediating anti-inflammatory effects. Both antioxidant and anti-inflammatory mechanisms are likely to be involved in the beneficial effects of NRF2 activation in NASH. Activation of NRF2 was also shown to limit the lipid accumulation in the liver of high-fat diet fed mice (7, 8). This effect has been suggested to be due to both inhibition of lipogenesis and stimulation of lipid oxidation in the liver.

NRF2 is primarily regulated at the posttranslational level *via* its binding partner KEAP1. Under basal conditions, Keap1 anchors Nrf2 in the cytoplasm and targets it for ubiquitination and consequently 26S proteasome mediated degradation. Keap1 functions as substrate receptor of a cullin-3–based E3 ubiquitin ligase. The Keap1 mediated constitutive ubiquitination and degradation represses the ability of Nrf2 to activate gene expression. Inducers of NRF2 are usually electrophilic compounds, which form adducts with specific cysteine residues in KEAP1. As a result, KEAP1 becomes inactivated and NRF2 accumulates and translocates into the nucleus, where it binds to the antioxidant response elements (AREs) of target genes.

Although various NRF2 activators have been developed and some have been used in clinical trials, there are concerns that long-term treatment may elicit adverse effects. For instance, NRF2 activation is known to promote tumorigenesis as well as mediate chemoresistance (9, 10). Inactivating mutations in KEAP1 or activating mutations in NRF2 are frequently found in a number of cancers, including cancers of the lung, gallbladder, and liver. While generally cytoprotective, NRF2 activators can also exert important other adverse effects. For instance, NRF2 activation has been reported to promote hypertension in diabetic mice *via* the transcriptional induction of angiotensin and angiotensin-converting enzyme in renal proximal tubule cells (11, 12). Nrf2 activation in regulatory T cells has been shown to promote regulatory T cell loss and to induce an autoinflammatory phenotype in mice (13). NRF2 has also been reported to play an important role in regulating hematopoietic stem cell quiescence (14, 15). In addition, electrophilic NRF2 activators are also likely to have off-target effects by reacting with other target proteins with reactive cysteine residues. These studies suggest that long-term treatment with NRF2 inducers can promote tumorigenesis and lead to unwanted side effects due to pleiotropic effects of NRF2 activation in different tissues and toxicity due to off target effects of electrophilic NRF2 activators. Indeed, a clinical trial for the treatment of patients with type 2 diabetes and chronic kidney disease with the triterpenoid Nrf2 activator bardoxolone methyl was interrupted prematurely because of an increased incidence of adverse cardiovascular events and death in drug recipients (16).

The goal of this study was to identify novel strategies to activate NRF2 for the treatment of NASH while avoiding tumor promoting effects as well as side effects in other tissues. We identified NRF2 inducer prodrugs that are activated in a cytochrome P450-dependent manner. We also identified the cellular oxygen concentration as a critical factor in determining the activity of NRF2-inducing drugs.

## Results

### tBHQ and 4-MC induce a robust activation of NRF2

**Figure 1:**
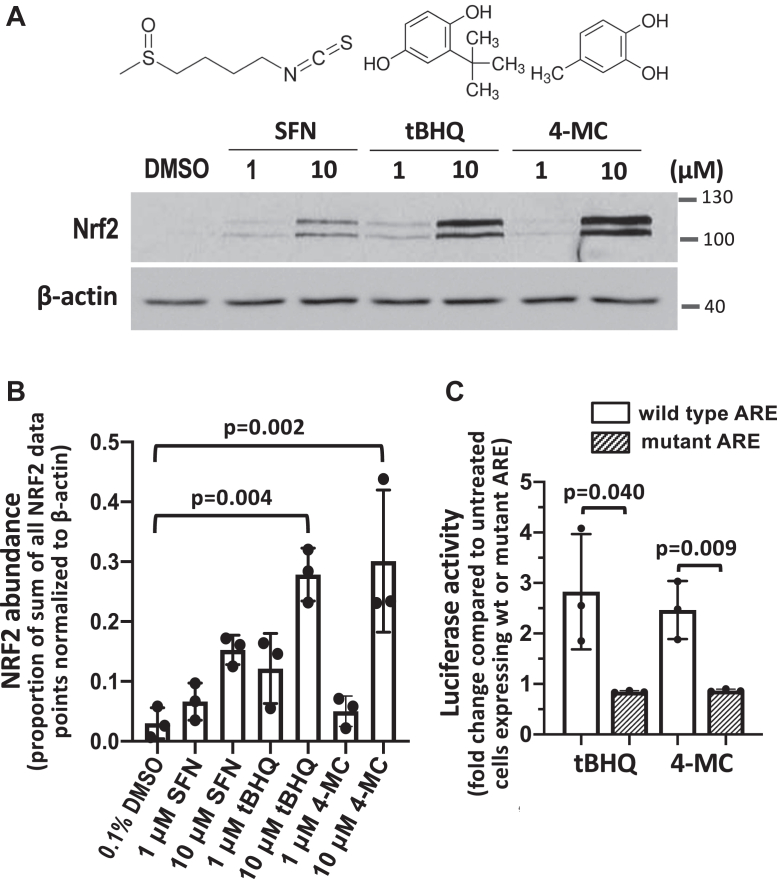
**Tert-butylhydroquinone and 4-methylcatechol activate NRF2 potently.***A,* HEK293T cells treated with sulforaphane (SFN), tBHQ, or 4-MC at the indicated concentrations (4 h), followed by measurement of NRF2 protein levels by Western blotting. *B,* the graph represents the mean and SD values from three independent Western blot experiments. The densitometry data were normalized by the sum of all data points method (40), as described under Experimental procedures. Statistical significance was evaluated using two-way ANOVA with Tukey's multiple comparisons test. *C,* NRF2 transcriptional activity in HEK293T cells was measured by transfecting cells with WT or mutant (inactive) ARE luciferase reporter plasmid for 40 h and treating the cells with 10 μM BHQ or 4-MC, as indicated, for the past 24 h, followed by determining the luciferase activity in the cell lysates. Data are mean ± SD of three independent experiments; *p* values are calculated using an unpaired *t* test. 4-MC, 4-methylcatechol; tBHQ, tert-butylhydroquinone; NRF2, nuclear factor-erythroid 2-related factor 2; ARE, antioxidant response element.

In order to explore liver selective NRF2 activator prodrugs that can be activated by cytochrome P450 enzymes *via* hydroxylation, we initially tested a small panel of NRF2 activator compounds whose molecular structures include one or more hydroxyl group(s). To assess the potency of NRF2 activation by these compounds, we used the NRF2 protein level in drug-treated HEK293T cells as a read-out of compound activity. Among the NRF2 activators tested, tert-butylhydroquinone (tBHQ) and 4-methylcatechol (4-MC) induced the highest level of NRF2 stabilization. When used at a concentration of 10 μM, both tBHQ and 4-MC caused a statistically significant increase in NRF2 protein concentrations (Fig. 1, *A* and *B*). Notably, both compounds were more potent in stabilizing NRF2 compared to the widely studied prototypical NRF2 activator sulforaphane (Fig. 1, *A* and *B*), consistent with a previous report (17).

To further validate the activity of both compounds with an independent assay, we measured NRF2 transcriptional activity using an NRF2 ARE luciferase reporter plasmid containing the ARE upstream of the *Nqo1* gene. NRF2 binding to the ARE transactivates luciferase gene expression and gives a read-out of NRF2 transcriptional activity. As a negative control, we also transfected the cells with a luciferase reporter plasmid in which the ARE sequence was mutated. At a concentration of 10 μM, both tBHQ and 4-MC induced an approximately 2.5-fold increase in ARE reporter activity while not stimulating luciferase activity in cells expressing the mutant ARE construct (Fig. 1*C*). These results suggest that tBHQ and 4-MC increase NRF2 transcriptional activity. Based on the Western blot and luciferase reporter results, tBHQ and 4-MC were considered promising candidates for the identification of prodrugs.

### Modifications of the hydroxyl group(s) in tBHQ and 4-MC convert the compounds into inactive NRF2 activator prodrugs

**Figure 2:**
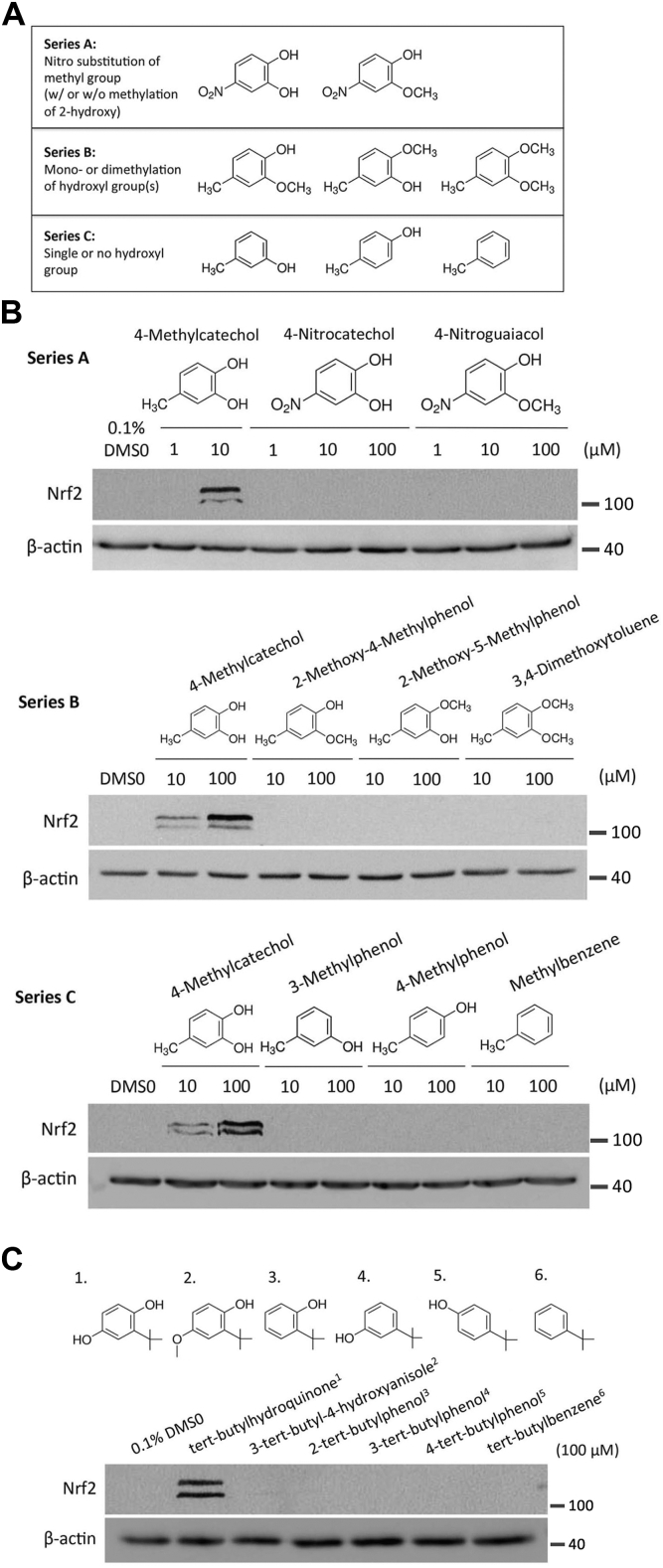
**Modifications of the hydroxyl groups on 4-MC and tBHQ abolished intrinsic NRF2-inducing activity.***A,* prodrug candidates of 4-MC. *B* and *C,* to determine the basal NRF2-inducing activity of prodrug candidates, HEK293T cells were treated for 4 h with 4-MC (*B*) and tBHQ (*C*) analogs, followed by measuring endogenous NRF2 protein concentrations by Western blotting. 4-MC, 4-methylcatechol; tBHQ, tert-butylhydroquinone; NRF2, nuclear factor-erythroid 2-related factor 2.

Both tBHQ and 4-MC contain functionally important hydroxyl groups that likely undergo tautomerization to form keto groups. The resulting benzoquinone structures contain an electrophilic α,β-unsaturated carbonyl, which can alkylate thiolates of reactive cysteines, for example, Cys151 in KEAP1, *via* a Michael addition reaction. This would lead to KEAP1 inhibition and NRF2 induction. To convert the active compound 4-MC into an inactive prodrug that can be activated by cytochrome P450 enzymes *via* hydroxylation, we selected prodrug analogs containing modifications that we hypothesized will result in reduced or abolished basal NRF2-inducing activity. These analogs either (1) possess one or both of the hydroxyl group(s) substituted with a methoxy group (Fig. 2*A*, series B) (2), lack one or both of the hydroxyl group(s) (series C) or have the 4-methyl group substituted with a nitro group (series A).

To determine the intrinsic NRF2-inducing activity of these prodrug analogs, we treated HEK293T cells that lack expression of cytochrome P450 enzymes with the prodrugs and measured the NRF2 protein level. No NRF2 activation was observed with all prodrug analogs from all three series when compared to the induction of NRF2 by the parental compound 4-MC (Fig. 2*B*), suggesting that these modifications completely abolished any intrinsic NRF2-inducing activity of the analogs. These analogs are therefore good starting points for prodrug candidates. Using a similar approach, we also selected prodrug analogs of tBHQ wherein one of the hydroxyl groups is substituted with a methoxy group or either one or both of the hydroxyl group(s) is/are absent. None of these prodrug analogs of tBHQ exerted any intrinsic NRF2-inducing activity even at a high concentration of 100 μM (Fig. 2*C*). The lack of intrinsic NRF2-inducing activity indicates that these tBHQ analogs are also good prodrug candidates.

### 3-Methylphenol, 2-tert-butylphenol, and 3-tert-butylphenol prodrugs can be activated by liver cytochrome P450 enzymes

**Figure 3:**
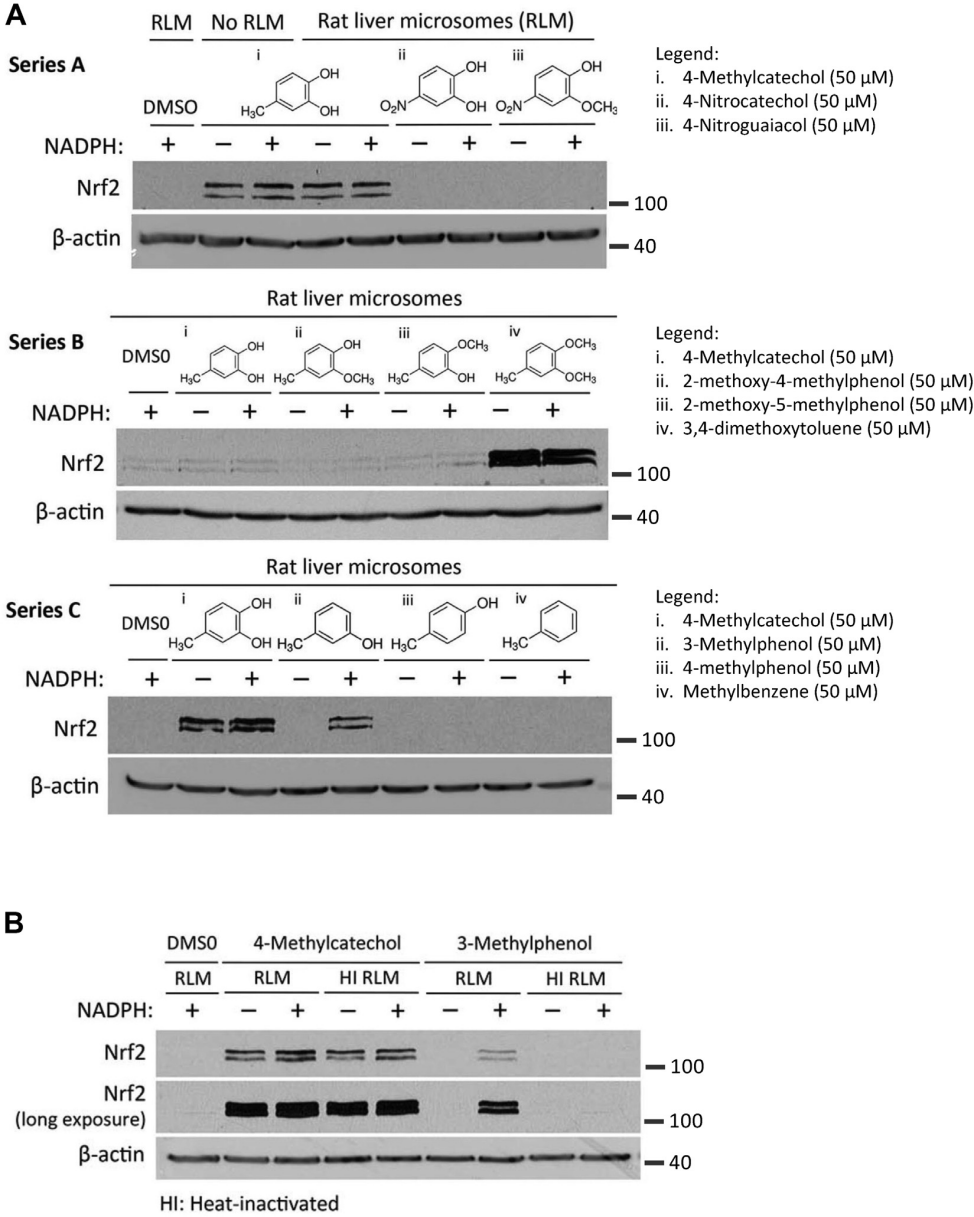
**The 3-methylphenol prodrug is activated by cytochrome P450 enzymes in rat liver microsomes.***A,* HEK293T cells were treated for 4 h with the indicated prodrug analogs of 4-MC after preincubation of the prodrug candidates with rat liver microsomes in the presence or absence of NADPH (1 mM, 1 h), as described under Experimental procedures. NRF2 protein levels were measured by Western blotting. *B,* the figure shows the NRF2 protein levels in HEK293T cells following treatment for 4 h with 4-MC or 3-methylphenol after preincubation of the compounds with functional (RLM) or heat-inactivated rat liver microsomes (HI RLMs) with or without NADPH (1 mM, 1 h). 4-MC, 4-methylcatechol; NRF2, nuclear factor-erythroid 2-related factor 2.

To next determine whether the prodrugs of 4-MC can be activated by liver cytochrome P450 enzymes, we preincubated the prodrugs with rat liver microsomes containing P450 enzymes, either in the presence or absence of NADPH cofactor that is required for P450 enzyme activity. Preincubated drugs were then added to HEK293T cells and NRF2 activation was assessed by measuring NRF2 protein expression levels. As shown in Figure 3*A*, one prodrug analog of 4-MC, 3-methylphenol with a single hydroxyl group at the meta-position, induced NRF2 in the presence but not in the absence of NADPH (series C). This suggests that 3-methylphenol can be activated by cytochrome P450 enzymes to induce NRF2. Notably, the prodrug analog 4-methylphenol, with a single hydroxyl group in the para-position, was not activated by rat liver microsomes and no induction of NRF2 was observed (series C). This suggests that cytochrome P450 enzymes exhibit some degree of substrate specificity. To confirm that activation of 3-methylphenol is dependent on cytochrome 450 enzyme activity, we preincubated the prodrug analogs with heat-inactivated rat liver microsomes, followed by treatment of HEK293T cells with the preincubated drug. No NRF2 induction was observed when 3-methylphenol was preincubated with the heat-inactivated rat liver microsomes in the presence of NADPH, confirming that activation of 3-methylphenol is indeed dependent on cytochrome P450 enzyme activity (Fig. 3*B*).

**Figure 4:**
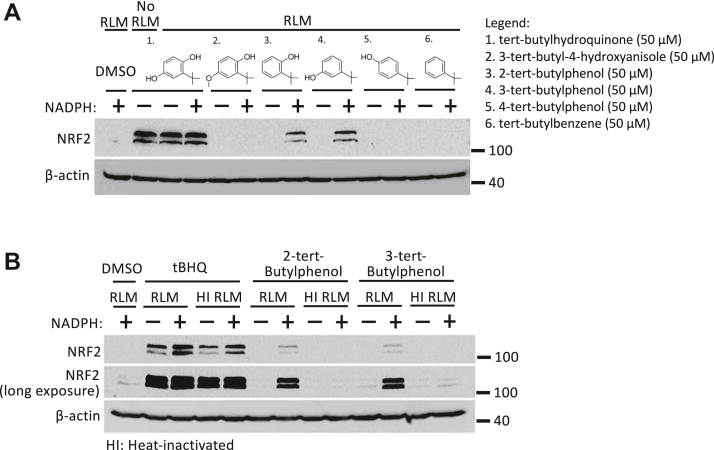
**Activation of 2-tert-butylphenol and 3-tert-butylphenol prodrugs by cytochrome P450 enzymes in rat liver microsomes.***A,* HEK293T cells were treated for 4 h with the indicated prodrug analogs of tBHQ after preincubation with rat liver microsomes (RLMs) with or without NADPH (1 mM, 1 h), as described under Experimental procedures. *B,* the figure shows the NRF2 protein levels in HEK293T cells following treatment with tBHQ, 2-tert-butylphenol, or 3-tert butylphenol (4 h) after preincubation with functional (RLM) or heat-inactivated rat liver microsomes (HI RLMs) with or without NADPH (1 mM, 1 h). 4-MC, 4-methylcatechol; tBHQ, tert-butylhydroquinone; NRF2, nuclear factor-erythroid 2-related factor 2.

Using the same bioassay, we examined tBHQ prodrug activation by rat liver microsomes. We identified two prodrug analogs of tBHQ, 2-tert-butylphenol and 3-tert-butylphenol, with a single hydroxyl group each at the 2- or 3-position that induced NRF2 in the presence but not in the absence of NADPH (Fig. 4*A*). This suggests that 2-tert-butylphenol and 3-tert-butylphenol can be activated by cytochrome P450 enzymes to induce NRF2. Further assessment using heat-inactivated rat liver microsomes showed loss in NRF2-inducing activities by both analogs, confirming that activation of 2-tert-butylphenol and 3-tert-butylphenol is dependent on cytochrome P450 enzymes (Fig. 4*B*).

**Figure 5:**
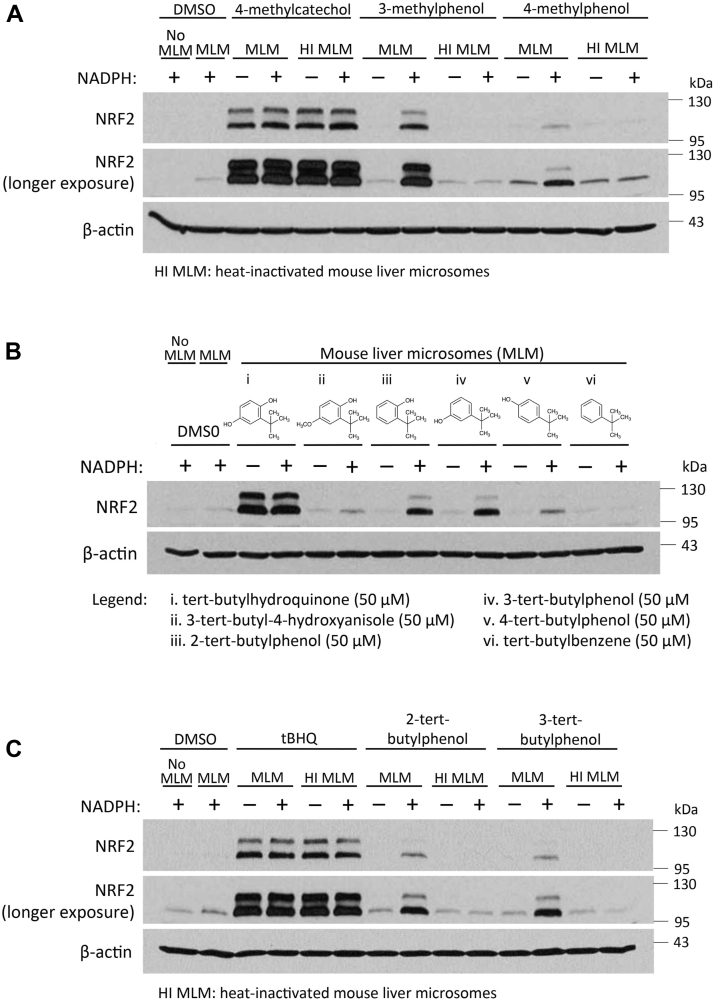
**Activation of 4-methylcatechol and tert-butylhydroquinone prodrugs by cytochrome P450 enzymes in mouse liver microsomes.***A*–*C,* HEK293T cells were treated for 4 h with the indicated prodrug analogs of 4-MC or tBHQ after preincubation with mouse liver microsomes (using functional (MLMs) or heat-inactivated mouse liver microsomes [HI MLMs]) with or without NADPH (1 mM, 1 h), as described under Experimental procedures. After the incubation, the cells were lysed and cell lysates subjected to Western blotting.

The cytochrome P450 homolog sequences and expression levels differ between rats and mice. Hence, we performed experiments analogous to Figures 3 and 4 using mouse liver microsomes. As shown in Figure 5, very similar results were obtained. Thus, 3-methylphenol, but not 4-methylphenol or any of the other potential 4-MC prodrugs shown in Figure 3*A*, induced NRF2 accumulation in HEK293T cells after preincubation with the mouse liver microsomes in the presence of NADPH (Fig. 5*A* and data not shown). Likewise, 2-tert-butylphenol and 3-tert-butylphenol, but not 4-tert-butylphenol, 3-tert-butyl-4-hydroxyanisol, and tert-butylbenzene, caused a notable increase in NRF2 expression in cells after preincubation with the mouse liver microsomes. Taken together, these results suggest that 3-methylphenol as well as 2-tert-butylphenol and 3-tert-butylphenol are prodrugs of 4-MC and tBHQ, respectively, which are activated *via* rat or mouse liver cytochrome P450 enzymes.

**Figure 6:**
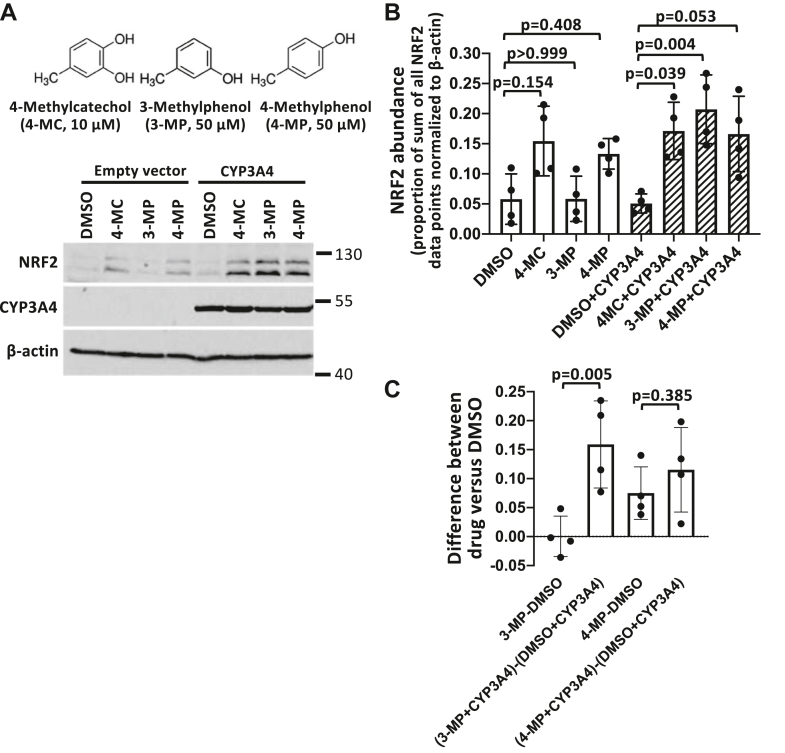
**CYP3A4 activates the prodrug 3-methylphenol.***A,* HEK293T cells were transfected with a CYP3A4 expression plasmid for 2 days, followed by 24 h treatment with 10 μM 4-MC and 50 μM of the prodrugs, as indicated, followed by Western blot analysis. *B,* the graph represents the mean and SD values from four independent Western blot experiments. The densitometry data were normalized by the sum of all data points method (40), as described under Experimental procedures. Statistical significance was evaluated using two-way ANOVA with Tukey's multiple comparisons test. *C,* the graph represents a statistical test of the differences in the prodrug effects in CYP3A4 transfected compared to control cells, which is necessary to determine whether the prodrugs have a selective effect in the presence of CYP3A4 (41). Thus, we calculated the differences in the NRF2 abundance, normalized to β-actin, of prodrug and DMSO-treated cells and compared these differences between empty vector and CYP3A4-transfected cells. The graph represents the mean and SD values from four independent Western blot experiments. Statistical significance was evaluated using an unpaired *t* test. 4-MC, 4-methylcatechol; NRF2, nuclear factor-erythroid 2-related factor 2.

Given that CYP3A4 is the most abundant cytochrome P450 homolog expressed in human liver and is responsible for metabolizing up to 50% of all known drugs in humans, we further examined whether activation of 3-methylphenol, 2-tert-butylphenol, and 3-tert-butylphenol can be mediated specifically by CYP3A4. To assess prodrug activation by CYP3A4, we utilized HEK293T cells, which normally lack CYP3A4 expression. We transfected the cells with a CYP3A4 expression plasmid or empty vector, followed by treatment of the cells with the active compound 4-MC or the prodrugs 3-methylphenol or 4-methylphenol and measurement of NRF2 activation by Western blotting. 4-MC induced NRF2 accumulation in both CYP3A4-transfected cells as well as in transfection control cells, although only the increase in CYP3A4-transfected cells reached statistical significance (Fig. 6, *A* and *B*). NRF2 induction by 4-MC in the presence and absence of CYP3A4 is expected because the drug does not require cytochrome P450-dependent activation. 4-methylphenol also caused an increase in NRF2 abundance in both the presence and absence of CYP3A4 (Fig. 6*A*). However, neither of these effects was statistically significant based on four independent Western blot experiments (Fig. 6*B*). In contrast, 3-methylphenol induced NRF2 in the CYP3A4-overexpressing cells to a level comparable to 4-MC, but was without effect in control cells (Fig. 6, *A* and *B*). Importantly, the difference in the effect of the 3-methylphenol prodrug in CYP3A4 transfected compared to control cells was highly significant (Fig. 6*C*). None of the other potential 4-MC prodrugs shown in Figure 3*A* induced a CYP3A4-dependent NRF2 accumulation in HEK293T cells (data not shown). Our results suggest that human CYP3A4 can mediate the activation of 3-methylphenol.

**Figure 7:**
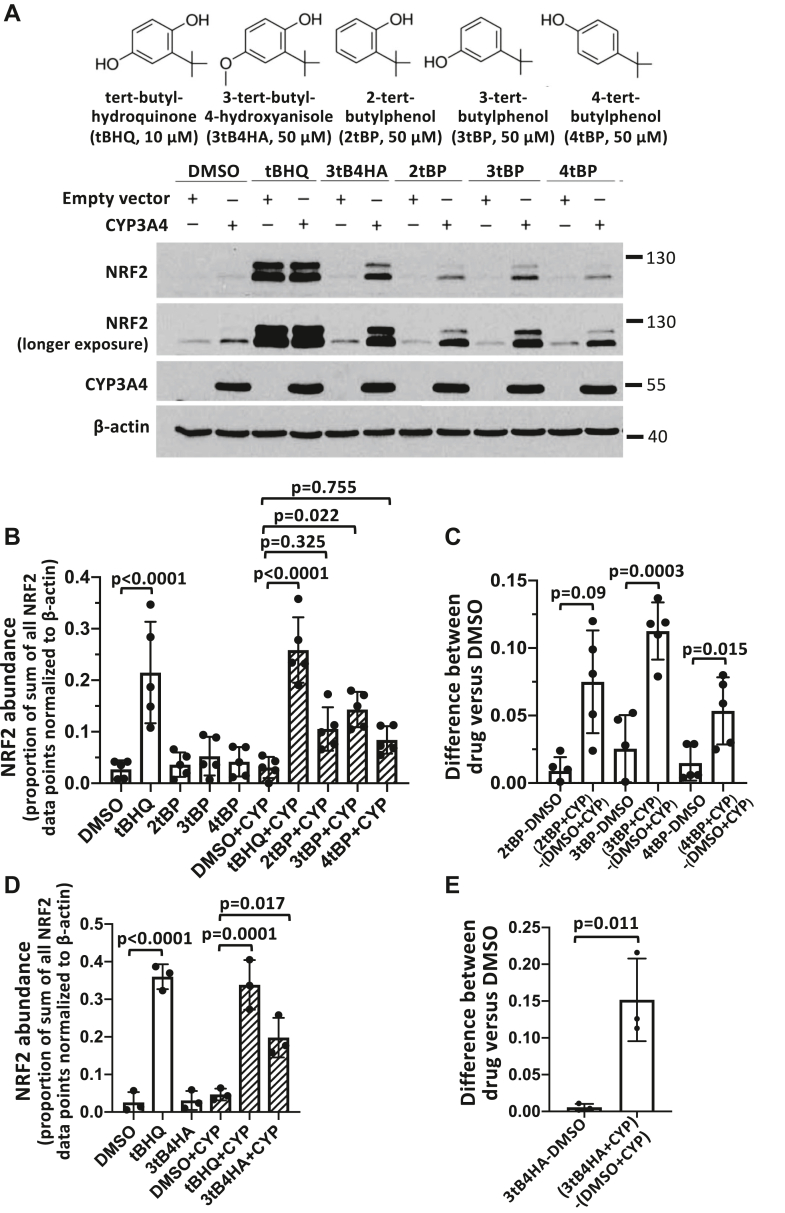
**CYP3A4 activates prodrugs 3-tert-butylphenol and 3-tert-4-hydroxyanisole.***A,* HEK293T cells were transfected with a CYP3A4 expression plasmid for 2 days, followed by 24 h treatment with 10 μM tBHQ and 50 μM of the prodrugs, as indicated, followed by measurement of NRF2 protein levels. *B* and *D,* the graph represents the mean and SD values from five (*B*) or three (*D*) independent Western blot experiments. The densitometry data were normalized by the sum of all data points method. Statistical significance was evaluated using two-way ANOVA with Tukey's multiple comparisons test. *C* and *E,* the graph represents a statistical test of the differences in the prodrug effects in CYP3A4-transfected cells compared to control cells. We calculated the differences in the NRF2 abundance, normalized to β-actin, of prodrug and DMSO-treated cells and compared these differences between empty vector and CYP3A4-transfected cells (*C*: n = 5; *E*: n = 4, *p* values calculated using an unpaired *t* test). tBHQ, tert-butylhydroquinone; NRF2, nuclear factor-erythroid 2-related factor 2.

We performed analogous experiments using the same CYP3A4 overexpression system with the tBHQ prodrug candidates. As expected, tBHQ induced a robust, highly statistically significant, increase in NRF2 protein expression in both CYP3A4 and empty vector transfected cells (Fig. 7, *A* and *B*). Consistent with the rat and mouse liver microsome results, 3-tert-butylphenol induced a statistically significant increase in NRF2 expression in CYP3A4-expressing HEK293T cells, but not in control cells (Fig. 7, *A* and *B*). The difference in the effect of the 3-tert-butylphenol prodrug in CYP3A4 transfected compared to control cells was also highly significant based on five independent experiments (Fig. 7*C*). However, the effect of 3-tert-butylphenol was smaller than the parental compound tBHQ. This suggests that CYP3A4 can catalyze activation of 3-tert-butylphenol, but may not represent the exclusive cytochrome P450 enzyme that is involved in this conversion. 2-tert-butylphenol, which was also activated by cytochrome P450 enzymes present in rat and mouse microsomes, displayed a similar trend to 3-tert-butylphenol (Fig. 7*A*). However, the effect of 2-tert-butylphenol was not statistically significant (Fig. 7, *B* and *C*). Notably, 4-tert-butylphenol and 3-tert-butyl-4-hydroxyanisole, which were not activated by rat and mouse liver microsomes, induced CYP3A4 dependent increases in NRF2 expression that were statistically significant (Fig. 7, *C* and *E*), while tert-butylbenzene did not cause any increase in NRF2 expression (data not shown). In particular, the NRF2 expression induced by 3-tert-butyl-4-hydroxyanisole in CYP3A4-expressing cells was pronounced (Fig. 7, *A* and *E*), suggesting that human CYP3A4 has different substrate specificities compared to its orthologs and paralogs in rat and liver microsomes. In conclusion, human CYP3A4 can catalyze the activation of 3-methylphenol, 3-tert-butylphenol, 4-tert-butylphenol, and 3-tert-butyl-4-hydroxyanisole. Among the various prodrugs, 3-methylphenol exhibited the most robust and consistent effects in the different experimental systems.

### 4-MC and tBHQ are inactive under hypoxic conditions

**Figure 8:**
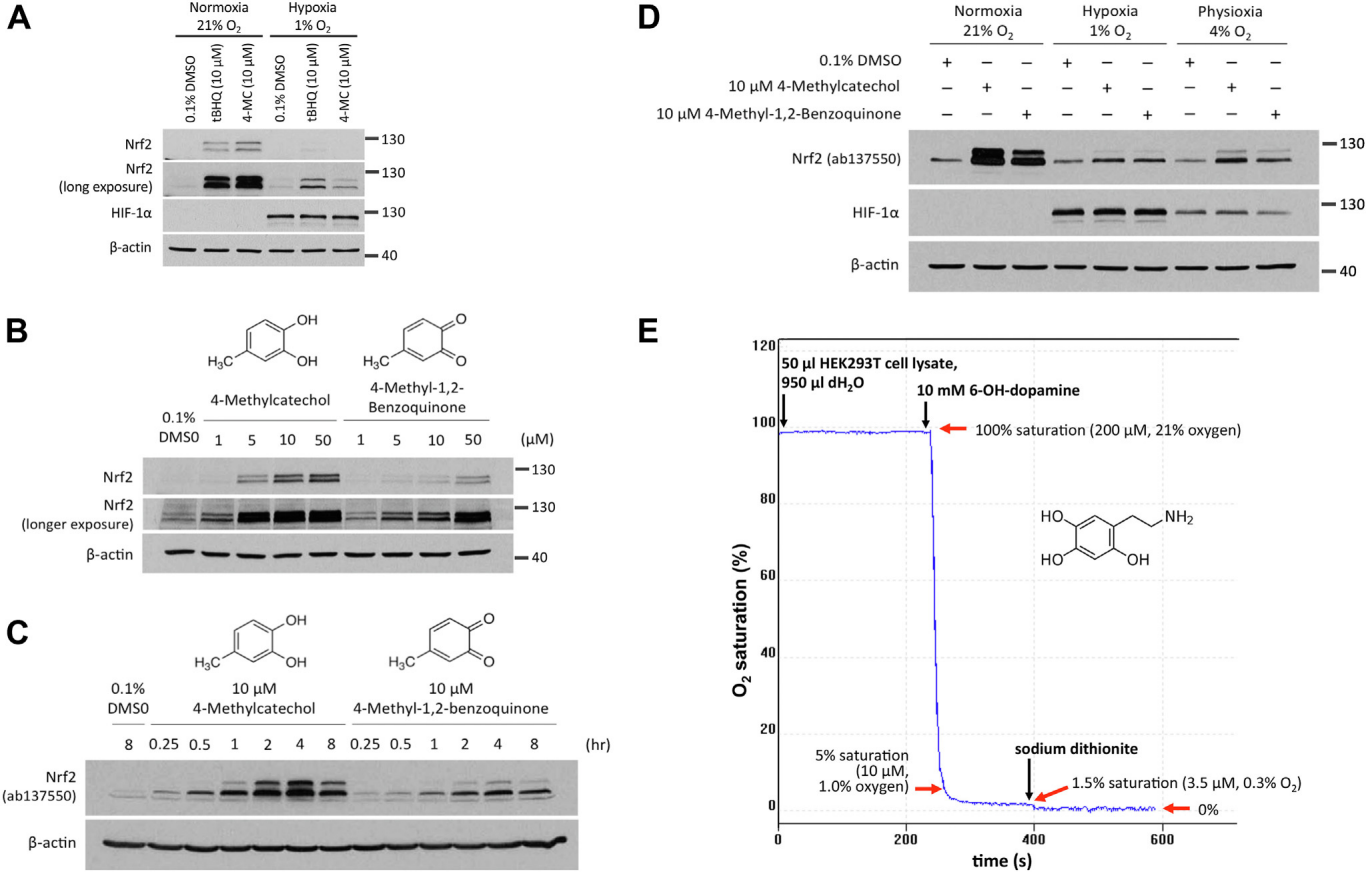
**4-MC and tBHQ are inactive under hypoxic conditions.***A,* HEK293T cells were treated with 4-MC and tBHQ for 4 h under normoxic (21% O_2_) or hypoxic (1% O_2_) conditions, as indicated, followed by measurement of NRF2 and HIF-1α protein levels. *B,* cells were treated with 4-MC and 4-methyl-1,2-benzoquinone for 4 h at increasing concentrations, as indicated, followed by measurement of NRF2 protein levels. *C,* cells were treated with 10 μM 4-MC and 4-methyl-1,2-benzoquinone for the indicated times, followed Western blotting for NRF2 protein levels. *D,* HEK293T cells were treated with 4-MC and 4-methyl-1,2-benzoquinone for 4 h at oxygen concentrations of 21%, 4%, or 1%, as indicated, followed by Western blotting for NRF2 and HIF-1α. *E,* autoxidation of 6-hydroxydopamine (6-OH-dopamine) was measured by monitoring oxygen consumption using a Clark type oxygen electrode. 6-OH-dopamine at a final concentration of 10 mM was added to the 1 ml chamber containing water and 50 μl of cell lysate (protein concentration of 2.5 μg/ml). Excess sodium dithionite was added where indicated in order to determine the remaining oxygen concentration in the chamber. 4-MC, 4-methylcatechol; tBHQ, tert-butylhydroquinone; NRF2, nuclear factor-erythroid 2-related factor 2; HIF-1α,hypoxia-inducible factor-1α.

Our results suggest that utilizing prodrugs of 4-MC and tBHQ may be a potential approach to selectively activate NRF2 in the liver and avoid nonspecific effects in other tissues. Of note, both 4-MC and tBHQ require bioactivation in an oxygen-dependent reaction to produce the reactive quinone compounds 4-methyl-1,2-benzoquinone and 2-tert-butyl-1,4-benzoquinone, respectively, which can then form adducts with Cys151 in KEAP1 (18). Hence, we investigated the oxygen dependence of the NRF2 activation by 4-MC and tBHQ, which could be an important factor for the efficacy as well as for the side effects of these compounds and our identified prodrugs in the context of the tumor environment. We exposed HEK293T cells to an oxygen concentration of 1% and treated the cells with tBHQ and 4-MC, followed by measuring NRF2 protein levels. As shown in Figure 8*A*, only a very small activation of NRF2 by 4-MC and tBHQ was observed at 1% oxygen when compared to the same treatment performed in cells at normoxia (oxygen concentration of 21%, which was utilized in all previous assays). This indicates that both 4-MC and tBHQ have a markedly reduced activity at low concentrations of oxygen, as found in the tumor environment.

To test whether the lack of activity of 4-MC and tBHQ in hypoxia is due to the requirement of oxygen for their conversion to the respective quinols, we utilized 4-methyl-1,2-benzoquinone, the oxidized active metabolite of 4-MC. As expected, 4-methyl-1,2-benzoquinone caused a dose-dependent and time-dependent increase in NRF2 protein expression (Fig. 8, *B* and *C*). When comparing the dose and time dependence of the NRF2 activation with that of 4-MC, 4-methyl-1,2-benzoquinone was found to exhibit a lower activity (Fig. 8, *B* and *C*). This may be due to the greater reactivity and consequently a shorter half-life of 4-methyl-1,2-benzoquinone.

We then compared the ability of 4-MC and 4-methyl-1,2-benzoquinone to induce NRF2 activation in hypoxia. Given that 4-methyl-1,2-benzoquinone does not require oxygen-dependent bioactivation, we expected that the compound would show a similar activity in both normoxia (21% oxygen) and hypoxia (1% oxygen). Unexpectedly, the activity of 4-methyl-1,2-benzoquinone was also dramatically inhibited in hypoxia (Fig. 8*D*). These results suggest that the low activity of 4-MC in hypoxia may not be due to the lack of bioactivation and that hypoxia may inhibit NRF2 activation by 4-MC and 4-methyl-1,2-benzoquinone *via* a different mechanism.

We also determined the effect of 4-MC and 4-methyl-1,2-benzoquinone at 4% oxygen, which is more representative of the physiological oxygen tension under *in vivo* conditions. As shown in Figure 8*D*, incubation of cells at 4% oxygen resulted in an intermediate stabilization of the Hypoxia-inducible factor-1α (HIF-1α) protein, as expected. Incubation at 4% also resulted in an intermediate induction of NRF2 by both 4-MC and 4-methyl-1,2-benzoquinone. Thus, low and physiological oxygen concentrations appear to inhibit 4-MC induced NRF2 stabilization independently of the oxygen-dependent bioactivation to the reactive quinone compound.

We next wanted to test if the oxygen-dependent oxidation of quinols to quinone compounds can still occur at oxygen concentrations of 4% and 1%. To this end, we measured the autoxidation of 6-hydroxydopamine, which is well known to undergo an autoxidation reaction analogous to catechols and hydroquinones to form a quinone compound. Autoxidation was measured by monitoring oxygen consumption using a Clark oxygen electrode. We found that the autoxidation rate of 6-hydroxydopamine was markedly increased in the presence of HEK293T cell lysate (data not shown). This may be due to the requirement of Cu^2+^ and possibly other divalent metal ions present in the cell lysate for the efficient autoxidation of 6-hydroxydopamine and 4-MC (19, 20, 21, 22). As shown in Figure 8*E*, addition of 10 mM 6-hydroxydopamine in the presence of cell lysate caused a decrease in the oxygen concentration, indicative of 6-hydroxydopamine autoxidation. The autoxidation reaction proceeded at a very fast rate to an oxygen concentration of 5% saturation, corresponding to 12 μM or 1.0% O_2_. 6-hydroxydopamine autooxidation continued at even lower oxygen concentrations, although at a slower rate, and ceased only at 1.5% oxygen saturation, corresponding to 3.5 μM or 0.3% O_2_. Addition of the reducing agent sodium dithionate, which reacts rapidly with oxygen, decreased the oxygen concentration in the chamber to 0. These results suggest that even in 1% hypoxia, autoxidation of 6-hydroxydopamine still occurs rapidly. Unfortunately, the rate of 4-MC oxidation was too slow to perform the same kind of analysis (data not shown). However, given the analogous autoxidation mechanism of 6-hydroxydopamine and 4-MC, it is likely that the oxygen dependence of the two compounds is similar. These results further support the conclusion that the inhibitory effect of hypoxia on NRF2 activation by 4-MC and tBHQ is not due to decreased bioactivation of these compounds.

### Inhibition of NRF2 activation by hypoxia is not limited to 4-MC and tBHQ

**Figure 9:**
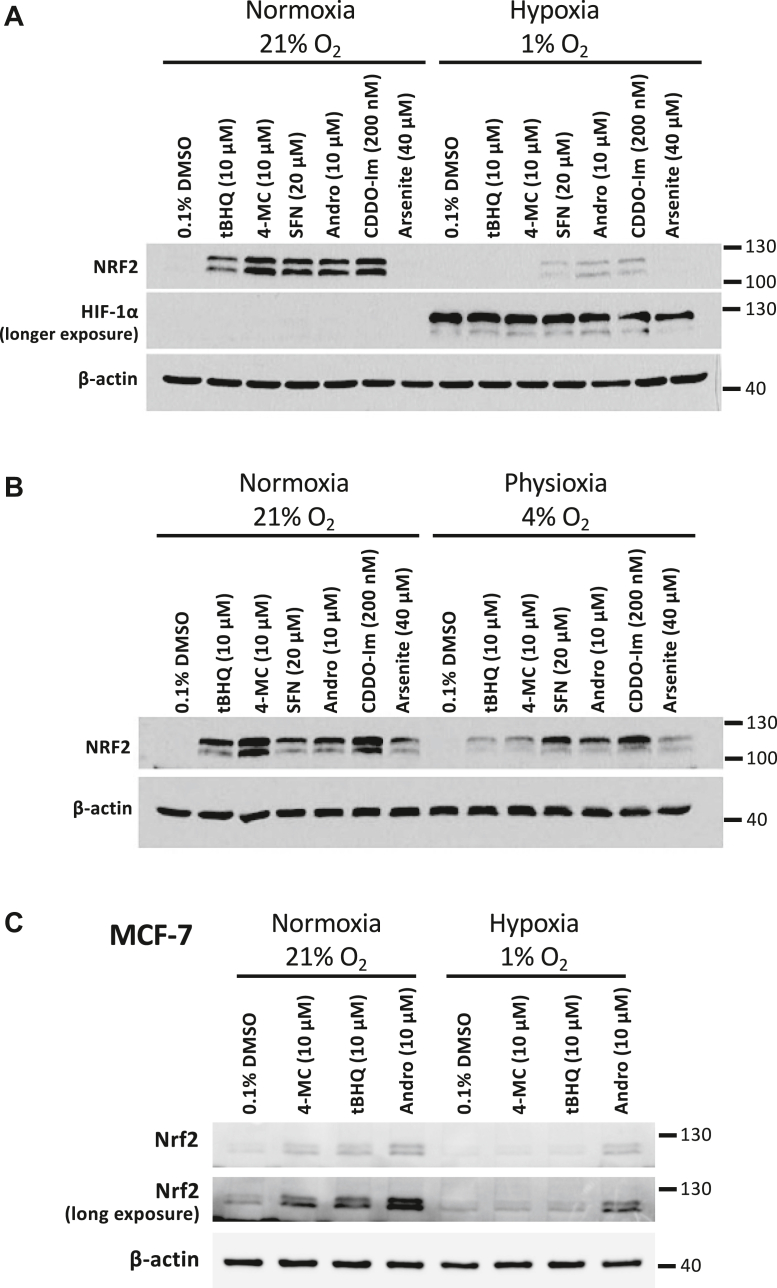
**Inhibition of NRF2 activation by hypoxia is not limited to 4-MC and tBHQ.***A* and *B,* HEK293T cells were treated for 4 h with the indicated drugs at 21%, 1%, or 4% oxygen (SFN = sulforaphane; Andro = andrographolide). Cell lysates were used for Western blotting with the indicated antibodies. *C,* MCF-7 cells were treated for 3 h with the indicated drugs at 21% or 1% oxygen, followed by Western blotting of cell lysates using NRF2 and β-actin antibodies. 4-MC, 4-methylcatechol; tBHQ, tert-butylhydroquinone; NRF2, nuclear factor-erythroid 2-related factor 2.

Given that the low activity of 4-MC and tBHQ in hypoxia is likely not a consequence of lack of autooxidation of these compounds, we tested whether hypoxia also affects the activity of other NRF2 inducing compounds. As shown in Figure 9*A*, the well-established NRF2 activators sulforaphane, andrographolide, and CDDO-imidazole (CDDO-Im) displayed a markedly reduced activity in hypoxia (1% oxygen). NRF2 activation by sulforaphane, andrographolide, and CDDO-Im was not inhibited at an oxygen concentration of 4% (Fig. 9*B*). In contrast, NRF2 induction by 4-MC and tBHQ was reduced at 4% oxygen compared to normoxia, although to a lesser degree than at an oxygen concentration of 1%. Arsenite caused no or only weak induction of NRF2 protein expression. As shown in Figure 9*C*, incubation of MCF-7 cells at 1% oxygen also resulted in less NRF2 induction by 4-MC, tBHQ, and andrographolide compared to incubation at 21% oxygen. In conclusion, hypoxia exerts an inhibitory effect on the stabilization of NRF2 by various NRF2 activators.

### The inhibitory effect of hypoxia on NRF2 is not due to activation of HIF-1**α** or inhibition of NRF2 protein synthesis

Given that the inhibitory effect of hypoxia on the NRF2 stabilization by various well-established NRF2 inducer compounds is likely highly relevant under *in vivo* conditions, in particular in hypoxic tumor tissue, we carried out mechanistic studies. Hypoxia leads to the stabilization of the transcription factor HIF-1α, which functions as a master regulator of the cellular hypoxic response by activating the transcription of numerous genes involved in the adaptation of cells to low oxygen concentrations. We hence first determined whether the inhibitory effect of hypoxia on NRF2 activation is mediated by HIF-1α. Treatment of HEK293T cells with the HIF-1α-inducing, hypoxia-mimetic compounds desferrioxamine and dimethyloxalylgycine resulted in an increase in HIF-1α protein expression (see V5 bot in Fig. S2*A*). However, desferrioxamine and dimethyloxalylgycine did not replicate the inhibitory effect of hypoxia on NRF2 induction in the presence of absence of transfected wild type HIF-1α (Fig.S1, *A and B*). These results argue against a role of HIF-1α.

**Figure 10:**
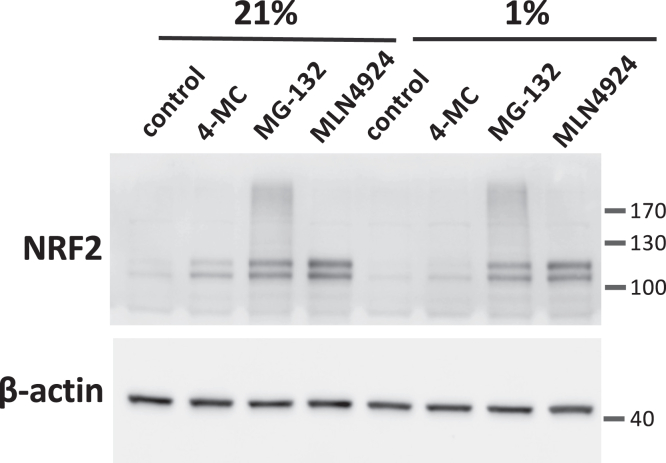
**The inhibition of drug-induced NRF2 accumulation at 1% oxygen is not due to an inhibitory effect of hypoxia on NRF2 synthesis.** HEK293T cells were treated for 3.5 h with 10 μM 4-MC, 10 μM MG-132, or 2 μM MLN-4924, as indicated. The cells incubated in hypoxia were pre-equilibrated at 1% oxygen for 30 min before drug addition and the drugs were added inside the hypoxic incubator. After the incubation time, the cells were lysed and the cell lysates were analyzed by Western blotting. 4-MC, 4-methylcatechol; NRF2, nuclear factor-erythroid 2-related factor 2.

We next determined whether the inhibition of drug induced NRF2 accumulation is due to an inhibitory effect on NRF2 synthesis or a faster protein degradation rate. Of note, hypoxia is well known to inhibit the cellular protein synthesis rate *via* inhibition of the mechanistic target of rapamycin complex I (mTORC1) activity as well as induction of eIF2α Ser51 phosphorylation. However, when NRF2 accumulation was induced by inhibiting its ubiquitination or proteasomal degradation using MLN4924 or MG-132, respectively, NRF2 protein accumulated to comparable levels in normoxia and hypoxia, suggesting that hypoxia does not inhibit NRF2 translation (Fig. 10). We also observed that inhibition of mTORC1 using Torin1, rapamycin or serum, and amino acid deprivation was without effect on drug-induced NRF2 accumulation in normoxia (Fig. S1, *B*–*D*). Although inhibiting mTORC1 with PP242 did prevent NRF2 induction (Fig.S1, *B* and *C*), the lack of effect of the other three approaches to inhibit mTORC1 suggests that the effect of PP242 is nonspecific. Furthermore, to determine if the effect of hypoxia is due to induction of eIF2α Ser51 phosphorylation, we overexpressed of a C-terminal deletion construct of GADD34, the regulatory subunit of protein phosphatase 1, which is highly effective in preventing eIF2α Ser51 phosphorylation (references (23, 24, 25) and Fig.S2*C*). As shown in Figure S2, *A* and *B*, preventing eIF2α Ser51 phosphorylation did not reverse the inhibitory effect of hypoxia on 4-MC and andrographolide-induced NRF2 accumulation. In conclusion, the inhibitory effect of hypoxia on NRF2 induction is unlikely due to lower NRF2 synthesis rates.

We also tested whether hypoxia affected the degradation rate of ubiquitinated NRF2. To this end, we first induced high NRF2 ubiquitination levels by treating cells for 4 h with the proteasome inhibitor MG-132. Subsequently, MG-132 was washed out by incubating cells with fresh medium, which contained the cullin E3 ligase inhibitor MLN4924 to prevent new NRF2 ubiquitination. The decrease in the levels of ubiquitinated NRF2 was then measured over time. We found that the rate with which ubiquitinated NRF2 decreased was comparable in normoxia and hypoxia (Fig.S2*D*). In conclusion, the inhibition of drug-induced NRF2 accumulation in hypoxia is unlikely due to an inhibitory effect on NRF2 synthesis or a faster protein degradation rate.

### The inhibitory effect of hypoxia on NRF2 is not due to a more reduced cellular redox state

**Figure 11:**
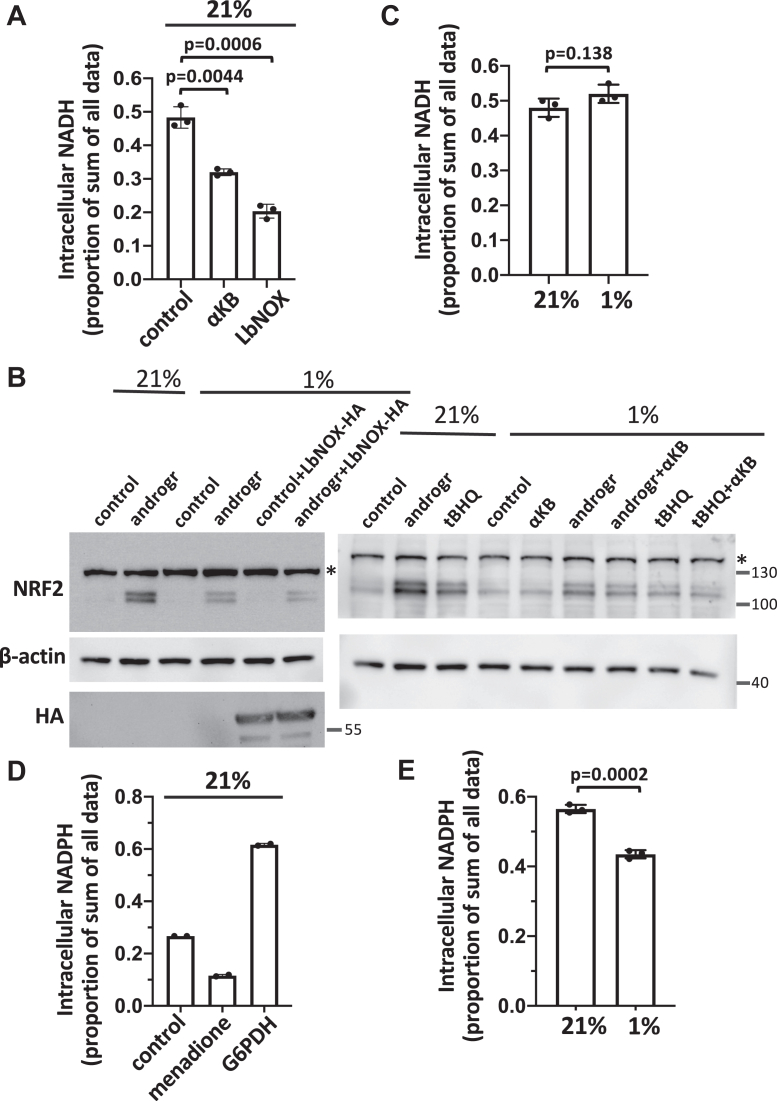
**The inhibition of drug induced NRF2 accumulation in hypoxia is not due to an increase in the cellular NADH or NADPH concentration.***A,* HEK293T cells were transfected for 2 days with an LbNOX expression plasmid or treated for 3 h with 2.5 mM α-ketobutyrate (αKB). The intracellular NADH concentrations were measured as described under Experimental procedures. The NADH luminescence readings were normalized to the protein concentration of the lysates and plotted according to the sum of all data points method (40), where each bar corresponds to the proportion of the sum of all luminescence readings. The graph represents the mean and SD values from three independent Western blot experiments. Statistical significance was evaluated using two-way ANOVA with Tukey's multiple comparisons test. *B,* the cells were transfected for 2 days with an LbNOX expression plasmid, where indicated, and treated for 3 h at 21% or 1% oxygen in the presence of 10 μM andrographolide, 10 μM tBHQ, and 2.5 mM α-ketobutyrate, as indicated. Subsequently, the cells were lysed and subjected to Western blotting. The asterisk in the NRF2 blots corresponds to a nonspecific band. *C,* HEK293T cells were incubated at 1% oxygen or kept at 21% for 4 h, followed by measurement of the cellular NADH concentration as in (*A*). The data were normalized and presented as described in (*A*). The graph represents the mean and SD values from three independent Western blot experiments. Statistical significance was evaluated using an unpaired *t* test. *D* and *E,* the cells were transfected with a human glucose-6-phosphate dehydrogenase (G6PDH) expression plasmid for 2 days and incubated at 21% oxygen in the presence of 80 μM menadione, as indicated, for 3 h (*D*) or incubated at 21% or 1% oxygen for 3 h (*E*). The intracellular NADPH concentrations were measured analogously to the NADH measurements in (*A*) and (*B*) using the NADP/NADPH-Glo Assay kit (Promega), as described under Experimental procedures. The data normalization and presentation was performed as in (*A*). The graphs in (*D*) and (*E*) represent the mean and SD values from two or three independent experiments, respectively. Statistical significance in (*E*) was evaluated using an unpaired *t* test. tBHQ, tert-butylhydroquinone; NRF2, nuclear factor-erythroid 2-related factor 2.

Given that we observed that treatment with electron transport chain inhibitors myxothiazol and sodium azide caused a partial inhibition of 4-MC–induced NRF2 stabilization (Fig.S1*C*), we hypothesized that hypoxia may inhibit cysteine oxidation and adduct formation in KEAP1 by increasing the cellular NADH concentration. Indeed, hypoxic conditions are generally considered to cause a more reduced cellular redox state as a consequence of lower electron transport chain activity and NADH accumulation. In order to test this hypothesis, we lowered the cellular NADH concentration in hypoxia using two approaches. First, we added α-ketobutyrate, which is transported into cells and then converted to nonmetabolizable α-hydroxybutyrate by β-hydroxybutyrate dehydrogenase in an NADH-consuming reaction (26, 27). Second, we overexpressed in the cytosol the *Lactobacillus brevis* NADH oxidase LbNOX as a genetic tool to decrease the cytosolic NADH concentration (28). As shown in Figure 11*A*, both interventions were effective in lowering the cellular NADH concentration. However, neither intervention reversed the inhibitory effect of hypoxia on drug-induced NRF2 accumulation (Fig. 11*B*). Consistent with these results, we found that hypoxia caused only a marginal increase in the cellular NADH concentration (Fig. 11*C*). Hence, it is unlikely that the inhibitory effect of hypoxia on NRF2 accumulation is due to an increased cellular NADH concentration.

We also considered the possibility that hypoxia may inhibit NRF2 accumulation by causing an increase in the cellular NADPH concentration, which would disfavor or reverse oxidative cysteine modifications. We first validated the NADPH assay by treating cells with the redox cycling drug menadione or by overexpressing the first enzyme of the phosphate pentose pathway glucose-6-phosphate dehydrogenase. As expected, these manipulations decreased or increased the cellular NADPH concentration, respectively (Fig. 11*D*). When we then incubated cells in hypoxia, we found that hypoxia causes a decrease in the cellular NADPH concentration (Fig. 11*E*), consistent with previous findings in hepatocytes (29). These results indicate that the inhibitory effect of hypoxia on NRF2 accumulation is not due to an increased cellular NADPH concentration.

### The inhibitory effect of hypoxia on NRF2 is due to slower kinetics of cysteine adduct formation in KEAP1

**Figure 12:**
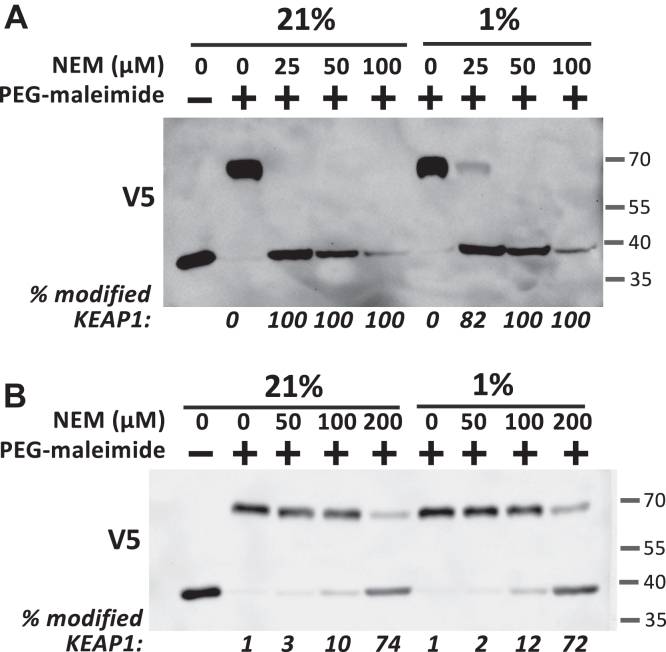
**Hypoxia inhibits drug-induced cysteine adduct formation in KEAP1.***A,* PEG-maleimide cysteine alkylation assay in HEK293T cells. The cells were transfected with the V5-KEAP1(amino acids 1–315)Cys151only-pcDNA3.1 plasmid, containing a single cysteine at position 151, for 2 days, followed by treatment of cells with the indicated concentrations of N-ethylmaleimide (NEM) for 90 min under conditions of 21% or 1% oxygen. The cells incubated in hypoxia were pre-equilibrated at 1% oxygen for 30 min prior to addition of NEM. After the incubation time, the cells were lysed in Triton X-100 containing lysis buffer in the absence of reducing agent. Aliquots of the cleared lysates were then mixed with an equal volume of 3 mM methoxypolyethylene glycol maleimide (PEG-maleimide) (molecular weight [MW] 10,000, Sigma-Aldrich Cat. No.712469) + 2% SDS in PBS (or in the absence of PEG-maleimide as negative control) for 20 min at room temperature. The PEG-maleimide alkylation reaction was stopped by adding 4x SDS loading buffer including 10% β-mercaptoethanol and analyzed by SDS-PAGE and Western blotting using a V5 antibody. The faster migrating V5 bands represent KEAP1(1–315) modified by NEM at Cys151. Absence of NEM modification resulted in alkylation of Cys151 by PEG-maleimide, leading to an increase in the MW and slower migration. The shown experiment is representative of four independent experiments (Fig. S3). *B, in vitro* PEG-maleimide cysteine alkylation assay. HEK293T cells grown in a Petri dish were transfected with the V5-KEAP1(amino acids 1–315)Cys151only-pcDNA3.1 plasmid for 2 days, followed by cell lysis in Triton X-100 containing lysis buffer in the absence of reducing agent. Aliquots of the cleared lysates were then incubated in the presence of the indicated NEM concentrations for 20 min and subsequently mixed with an equal volume of 3 mM PEG-maleimide + 2% SDS in PBS for 20 min at room temperature. The PEG-maleimide alkylation reaction was stopped by adding 4x SDS loading buffer including 10% β-mercaptoethanol and analyzed by SDS-PAGE and Western blotting using a V5 antibody.

We finally investigated the hypothesis that hypoxia inhibits drug-induced cysteine adduct formation in KEAP1. Toward this end, we measured NRF2 adduct formation in cells directly using a Western blot based PEG-maleimide cysteine alkylation assay. Thus, we transfected cells with a truncated V5-epitope–tagged KEAP1 plasmid, lacking the double-glycine repeat domain responsible for substrate binding. Given that Cys151 is the main target of NRF2-inducing drugs, we used a plasmid in which all cysteines except Cys151 were mutated to serine (V5-KEAP1(1–315)Cys151only). Cells were treated with increasing concentrations of the cysteine alkylating agent N-ethylmaleimide (NEM) at 21% or 1% oxygen, followed by cell lysis under non-reducing conditions and treatment of the cell lysates with methoxypolyethylene glycol-maleimide (PEG-maleimide, molecular weight 10,000 g/mol). Only non-modified Cys151 reacts with PEG-maleimide, resulting in an approximately 30 kDa band shift in a Western blot. As shown in Figure 12*A*, treatment of cells with increasing NEM concentrations resulted in a gradual disappearance of the high molecular weight, PEG-maleimide modified KEAP1 band, indicating alkylation of KEAP1 at Cys151 in cells. We conducted four independent experiments. Because there was substantial inter-experiment variation in the NEM concentrations required to induce Cys151 alkylation, we were unable to carry out a quantitative analysis. However, we found a right shift in the NEM adduct formation concentration dependence in hypoxia in all four repeats (Fig. S3). In conclusion, hypoxia appears to exert an inhibitory effect on KEAP1 Cys151 modification, resulting in lower kinetics of KEAP1 inhibition and NRF2 stabilization.

Finally, we also compared the NEM-induced modification of Cys151 in KEAP1 in lysates of cells transfected with the V5-KEAP1(1–320)Cys151only plasmid in normoxia *versus* hypoxia using the same experimental approach. Of note, we found that under these *in vitro* conditions hypoxia had no inhibitory effect (Figs. 12*B*, S4), suggesting that a cellular mechanism is involved in the oxygen dependent regulation of KEAP1 adduct formation.

## Discussion

NRF2 plays a central role in maintaining redox homeostasis in health and disease through its antioxidant and cytoprotective capacities. Activation of NRF2 by cysteine electrophiles that inhibit KEAP1 has been extensively studied as a therapeutic approach in diverse disease contexts including NAFLD, where oxidative stress and inflammation drive disease pathologies (30). A major limitation to the effective translation of NRF2 activators to the clinic lies in their poor safety profiles due to *in vivo* side effects including their potential tumor promoting properties. These effects of NRF2 activators are of special concern given that any treatment of NASH or NAFLD would likely be chronic and it is often difficult to anticipate patient risk factors such as cancer susceptibility. Therefore, we sought to explore novel therapeutic approaches that enable activation of NRF2 selectively in the liver to target the pathological triggers of NAFLD and NASH without initiating off-target tissue effects.

To initially explore potential prodrug candidates of 4-MC and tBHQ that can be activated in a cytochrome P450-dependent, liver selective manner, we tested two possible approaches. In the first approach, we used methoxy analogs of 4-MC and tBHQ. This approach was inspired by a report by Perry and colleagues (31), who demonstrated that demethylation of a methoxy group is an effective approach for achieving liver selective prodrug activation of the mitochondrial uncoupler 2,4-dinitrophenol *via* cytochrome P450 enzymes. Furthermore, Fetherrolf *et al.* (32) have shown that members of the cytochrome P450 CYP255A1 and CYP255A2 families from the bacterial strains *Rhodococcus rhodochrous* EP4 and *Rhodococcus jostii* RHA1 can mediate the efficient O-demethylation of 4-alkylguaiacols to 4-alkylcatechols (including that of 2-methoxy-4-methylphenol to 4-MC). Second, we used analogs of 4-MC and tBHQ that lack one or both phenolic hydroxyl groups. The latter approach proved to be more successful, and we identified two analogs of 4-MC and tBHQ, 3-methylphenol and 3-tert-butylphenol, respectively, that lack one hydroxyl group and are activated *via* cytochrome P450-mediated hydroxylation in rat, mouse, and human experimental systems. These studies provide proof-of-principle of a liver cytochrome P450 enzymes–dependent NRF2 prodrug activation approach. We believe that this approach can be leveraged to facilitate a high local drug concentration in the liver and mitigate NRF2 effects in off-target tissues *in vivo*.

We also observed that both tBHQ and 4-MC are inactive or markedly less active under hypoxic conditions (1% oxygen). This finding is consistent with one previous report by Senger *et al.* (17), who found lower activity of the NRF2 activators 4-MC and 4-ethylcatechol at an oxygen concentration of 2% compared to 21%. Notably, the physiological oxygen concentrations at which NRF2 activators exert their effects *in vivo* and especially the oxygen concentrations in tumors are much lower than those commonly employed in cell culture experiments. Hence, the markedly reduced activity of 4-MC is of great importance. Therefore, we conducted experiments to study the underlying mechanism.

Both 4-MC and tBHQ are quinol compounds, which initially undergo an oxygen-dependent autooxidation reaction. This gives rise to the respective quinones, which then form adducts with reactive cysteines *via* Michael addition. We therefore considered the possibility that the oxygen dependence of 4-MC and tBHQ is due to the requirement to undergo autooxidation. However, several reasons argue against this hypothesis. First, the activated form of 4-MC, 4-methyl-1,2-benzoquinone, exhibits a similar oxygen dependence as compared to the parent compound. Second, the oxidation of the quinol 2-hydroxydopamine to its quinone form in the presence of cell lysate can still occur efficiently at 1% oxygen. Finally, other NRF2 activator compounds, including sulforaphane, andrographolide, and CDDO-Im, which do not require activation *via* autooxidation, also exhibit lower NRF2-inducing activity in hypoxia.

Our results indicate that the low NRF2 activation by 4-MC, andrographolide, and potentially other NRF2 activators in hypoxia is not mediated through HIF-1α stabilization or mTORC1 inhibition. Hypoxia is also known to cause a more reduced cellular redox state, which may promote the inactivation of electrophilic NRF2 activators or antagonize oxidative cysteine modifications. However, our results suggest that the inhibition of the NRF2 response in hypoxia is independent of the cellular NADH and NADPH concentrations. Instead, our results suggest that the inhibitory effect of hypoxia on NRF2 is due to slower kinetics of cysteine adduct formation in KEAP1. Notably, this effect was only observed in intact cells, but not under *in vitro* conditions. It remains to be determined how hypoxia inhibits cysteine reactivity. One potential mechanism is that hypoxia lowers the sensitivity of KEAP1 cysteine residues to become modified due to a lower cellular pH, resulting in a lower proportion of cysteines existing in the reactive cysteine thiolate anion state. However, we found that varying the pH in the cell culture media between 6.6 and 8.0 or increasing the intracellular pH inside the cells through treatment with 10 mM NH_4_Cl did not affect drug-induced NRF2 stabilization (data not shown), arguing against this mechanism.

Irrespective of the precise mechanism through which hypoxia inhibits the effect of various NRF2 activators, the results of this study have important implications. First, they suggest that NRF2 activators might have very low activity under conditions of tumor hypoxia. Thus, tumor-promoting effects during long-term treatment with NRF2 activators may be a smaller concern when considering long-term treatment of chronic diseases such as NAFLD and NASH with NRF2 activator drugs. Second, the results of our study imply that it is important to take into account the effect of the oxygen concentration when evaluating any new NRF2-inducing compounds and to assess their effects at low oxygen concentrations found in tumors and at physiological oxygen concentrations present in tissues *in vivo*.

A large body of research has demonstrated the beneficial effects of NRF2 activation in several NAFLD and NASH related clinical metrics including hepatic steatosis, inflammation, and insulin resistance (33). While various recent studies have confirmed the beneficial role of NRF2 in NAFLD and NASH (34, 35, 36), there has also been a recent report suggesting that NRF2 activation in the liver does not lead to overall metabolic improvement but can cause liver pathology and systemic metabolic disturbances (37). The authors reported that NRF2 activation as a result of hepatocyte-specific knockout of cullin 3 results in rapid resolution of steatosis in obese mice, confirming various other studies. The resolution of steatosis was due to NRF2-dependent inhibition of lipogenic genes and induction of lipolytic genes, as well as induction of NADH reductive stress. However, resolving steatosis in hepatocyte Cul3 KO mice did not lead to overall metabolic improvement but caused liver pathology and systemic metabolic changes, including hepatic ceramide accumulation, elevated circulating fatty acid levels, and systemic insulin resistance. The authors hypothesized that this phenotype is a consequence of an NRF2-induced triglyceride storage defect, increased lipolysis, and inhibition of hepatic glucose oxidation.

Irrespective of the still uncertain therapeutic potential of liver selective NRf2 activation in mice and particularly in humans, we believe that our findings are of significance for a number of reasons. First, they provide important insights about how cytochrome P450 enzymes can be leveraged to achieve liver selective prodrug activation. Second, our study identifies NRF2 activation *via* the formation of cysteine adducts in KEAP1 as an oxygen-regulated process and characterizes the oxygen dependence of this process. These findings are of fundamental importance for our understanding of NRF2 biology, to predict and assess therapeutic effects of NRF2 activators as well as for the discovery and evaluation of new NRF2 activator drugs.

## Experimental procedures

### Cell culture

HEK293T cells were obtained from the ATCC (CRL-3216) and were cultured in Dulbecco's modified eagle medium (Invitrogen) supplemented with 10% (v/v) heat-inactivated fetal bovine serum (Hyclone), 2 mM L-glutamine (Invitrogen), 100 U/ml penicillin, and 100 μg/ml streptomycin (Invitrogen) in a humidified 37 °C, 5% CO2 tissue culture incubator. For hypoxia incubations, HEK293T cells in 12-well plates were pre-equilibrated under controlled oxygen partial pressure of 1% or 4% (5% CO_2_, 94% or 91% N_2_, respectively) in a humidified 37 °C hypoxia workstation (Coy Laboratory Products, O_2_ Control Polymer Glove Box) for 1 h and treated with the different NRF2 activators or 0.1% DMSO for 4 h. After treatment, cells were lysed inside the hypoxia workstation followed by Western blot analysis. For normoxic conditions, cells were treated similarly and incubated in a humidified 37 °C, 5% CO_2_ tissue culture incubator for 4 h.

### ARE luciferase reporter assay

The ARE-pGL2 reporter plasmid contained the 25-bp sequence (GCAGTCACAGTGACTCAGCAGAATC) of the ARE upstream of the *Nqo1* gene (NCBI# M81596), containing the core ARE 5′GTGACNNNGC′3′ (38), cloned into the KpnI and XhoI restriction sites of the pGL2-promoter plasmid (Promega). To generate the mutant ARE-pGL2 plasmid, the core ARE sequence was mutated to 5′AAAAANNNAA′3′. Thus, the 25-bp mutant sequence corresponding to GCAGTCACA**AAA**A**A**TCA**AA**AGAATC was inserted between the KpnI and XhoI restriction sites. HEK293T cells in 12-well plates at 50% confluency were transfected (Genejuice, Merck Millipore) with 0.1 μg of WT or mutant ARE-pGL2 plasmid for 40 h and treated with 1 μM or 10 μM of tBHQ or 4-MC, 10 μM of sulforaphane, or 0.1% DMSO for the last 24 h. Cells were lysed directly in 80 μl of Steady-Glo Reagent (E2510, Promega), incubated for 5 min at room temperature, and the luminescence in 25 μl aliquots of cell lysates was measured using a luminometer.

### Western blotting

Cells were washed with ice-cold PBS and lysed in 100 μl of Triton-lysis buffer (25 mM Tris–HCl, pH 7.5, 100 mM NaCl, 2.5 mM EDTA, 2.5 mM EGTA, 20 mM NaF, 1 mM Na3VO4, 20 mM sodium β-glycerophosphate, 10 mM sodium pyrophosphate, 0.5% Triton X-100, Roche protease inhibitor cocktail, and 0.1% β mercaptoethanol). Lysates were precleared by centrifugation at 16,000*g*, 5 min, 4 °C, denatured and reduced with Laemmli sample buffer containing 5% β-mercaptoethanol, and boiled at 95 °C for 10 min. Equal amounts of proteins were resolved on 10% SDS-PAGE gels and transferred onto nitrocellulose membranes. The membranes were immunoblotted with anti-NRF2 (Cell Signaling, C12721, rabbit monoclonal or Abcam, ab137550, rabbit polyclonal), mouse anti-HIF-1α (BD Pharmingen, 610959), anti-β-actin (Sigma, A5316, mouse monoclonal), anti-CYP3A4 (Santa Cruz, sc-53850, mouse monoclonal), anti GAPDH (US Biological, G8140-04, mouse monoclonal or Santa Cruz, sc-365062, mouse monoclonal), anti-phospho p70 S6K1 (Cell Signaling, 9234, rabbit monoclonal), anti-phospho 4EBP1 (Cell Signaling, 2855, rabbit monoclonal), anti HA (Roche, clone 3F10, rat monoclonal), and anti-V5 (AbD Serotec, MCA1360, mouse monoclonal) antibodies, followed by incubation with horseradish peroxidase–conjugated secondary antibodies. Enhanced chemiluminescence detection was carried out with Immobilon Western Chemiluminescent horseradish peroxidase substrate (Millipore) using the Bio-Rad ChemiDoc MP Imaging system operated in the signal accumulation mode. All shown Western blot results are representative of at least two independent experiments.

### Prodrug activation bioassay

All prodrugs of 4-MC and tBHQ (1 mM) or DMSO (0.25%) were preincubated with pooled male and female rat liver microsomes (0.75 mg microsomal protein per ml, M9066 and M9191, Sigma) or mouse liver microsomes (M9441 and M9566, Sigma) in PBS at 37 °C in a shaking water bath for 5 min. The reaction was initiated with 1 mM NADPH or without NADPH as control and incubated for an additional 1 h (total reaction volume of 200 μl). Microsomal suspensions were then centrifuged at 16,000*g* for 10 min at 4 °C to pellet the microsomes. The supernatant (50 μl) was then added to 90 to 95% confluent HEK293T cells (final drug concentration of 50 μM) in 12-well plates and incubated at 37 °C, 5% CO_2_ for 4 h, followed by cell lysis and Western blot analysis. For assays where heat-inactivated microsomes were used, pooled microsomes in PBS were heated at 95 °C for 10 min and cooled to room temperature before incubation with the prodrugs.

### CYP3A4 overexpression

Human CYP3A4 overexpression plasmid was generated by gene amplification from pCellFree_G03 CYP3A4 (a gift from Kirill Alexandrov, Addgene plasmid # 67104) (39) and cloning of the PCR product into pcDNA3 (using 5′ KpnI and 3′ XbaI restriction sites). The sequence was verified by Sanger sequencing. HEK293T cells in 12-well plates at 50% confluency were transfected (Genejuice, Novagen) with 0.8 μg CYP3A4 pcDNA3 or empty pcDNA3 for 36 to 52 h, and treated with 10 μM of tBHQ or 4-MC, or 50 μM of the different prodrug candidates for the last 24 h. Cells were then lysed, followed by Western blot analysis.

### Measurement of the intracellular NADH and NADPH concentrations

Following various cell treatments in 24-well plates, the cell culture media was removed and the cells rinsed with PBS and lysed by adding 100 μl PBS and 100 μl base solution (0.2 N NaOH, 0.5% Triton X-100). Aliquots of the cleared lysate were heated for 15 min at 60 °C to degrade intracellular NAD^+^ and neutralized by adding an equal volume of 0.4 N HCl/0.5 M Trizma base (pH10.7) solution, followed by the measurement of the intracellular NADH concentration using the NAD/NADH-Glo Assay kit (Promega), according to the manufacturer's instructions. The luminescence readings were normalized to the protein concentration of the lysates. The intracellular NADPH concentration was determined with the NADP/NADPH-Glo Assay kit (Promega) by using the protocol for cell lysis and NADP+ degradation as described for the NADH measurements.

### Statistical analysis

The Western blot densitometry data were normalized by the sum of all data points method (40). After performing densitometry analysis for Nrf2 and the β-actin loading control using the ImageJ 1.50i software application and calculating the Nrf2/β-actin ratios, all Nrf2/β-actin ratios (control and all treatments) were added up. For each sample Nrf2/β-actin ratio, we then calculated the proportion of the total Nrf2/actin ratio sum. Statistical significance was analysed using two-way ANOVA and Tukey's multiple comparisons test or unpaired *t* tests, as indicated in the figure legends.

## Data availability

All data generated are contained within the article. Data and material requests should be made to T. H.

## Supporting information

This article contains supporting information.

## Conflict of interests

The authors declare that they have no conflicts of interest with the contents of this article.

## References

[1] Targher G., Day C.P., Bonora E. (2010). Risk of cardiovascular disease in patients with nonalcoholic fatty liver disease. N. Engl. J. Med..

[2] Williams K.H., Shackel N.A., Gorrell M.D., McLennan S.V., Twigg S.M. (2013). Diabetes and nonalcoholic fatty liver disease: a pathogenic duo. Endocr. Rev..

[3] Söderberg C., Stål P., Askling J., Glaumann H., Lindberg G., Marmur J. (2010). Decreased survival of subjects with elevated liver function tests during a 28-year follow-up. Hepatology.

[4] Sugimoto H., Okada K., Shoda J., Warabi E., Ishige K., Ueda T. (2010). Deletion of nuclear factor-E2-related factor-2 leads to rapid onset and progression of nutritional steatohepatitis in mice. Am. J. Physiol. Gastrointest. Liver Physiol..

[5] Okada K., Warabi E., Sugimoto H., Horie M., Gotoh N., Tokushige K. (2013). Deletion of Nrf2 leads to rapid progression of steatohepatitis in mice fed atherogenic plus high-fat diet. J. Gastroenterol..

[6] Axelsson A.S., Tubbs E., Mecham B., Chacko S., Nenonen H.A., Tang Y. (2017). Sulforaphane reduces hepatic glucose production and improves glucose control in patients with type 2 diabetes. Sci. Transl. Med..

[7] Tanaka Y., Aleksunes L.M., Yeager R.L., Gyamfi M.A., Esterly N., Guo G.L. (2008). NF-E2-Related factor 2 inhibits lipid accumulation and oxidative stress in mice fed a high-fat diet. J. Pharmacol. Exp. Ther..

[8] Yang G., Lee H.E., Lee J.Y. (2016). A pharmacological inhibitor of NLRP3 inflammasome prevents non-alcoholic fatty liver disease in a mouse model induced by high fat diet. Sci. Rep..

[9] Krall E.B., Wang B., Munoz D.M., Ilic N., Raghavan S., Niederst M.J. (2017). KEAP1 loss modulates sensitivity to kinase targeted therapy in lung cancer. Elife.

[10] Zheng A., Chevalier N., Calderoni M., Dubuis G., Dormond O., Ziros P.G. (2019). CRISPR/Cas9 genome-wide screening identifies KEAP1 as a sorafenib, lenvatinib, and regorafenib sensitivity gene in hepatocellular carcinoma. Oncotarget.

[11] Abdo S., Shi Y., Otoukesh A., Ghosh A., Lo C.S., Chenier I. (2014). Catalase overexpression prevents nuclear factor erythroid 2-related factor 2 stimulation of renal angiotensinogen gene expression, hypertension, and kidney injury in diabetic mice. Diabetes.

[12] Zhao S., Ghosh A., Lo C.S., Chenier I., Scholey J.W., Filep J.G. (2018). Nrf2 deficiency upregulates intrarenal angiotensin-converting enzyme-2 and angiotensin 1-7 receptor expression and attenuates hypertension and nephropathy in diabetic mice. Endocrinology.

[13] Klemm P., Rajendiran A., Fragoulis A., Wruck C., Schippers A., Wagner N. (2019). Nrf2 expression driven by Foxp3 specific deletion of Keap1 results in loss of immune tolerance in mice. Eur. J. Immunol..

[14] Tsai J.J., Dudakov J.A., Takahashi K., Shieh J.H., Velardi E., Holland A.M. (2013). Nrf2 regulates haematopoietic stem cell function. Nat. Cell Biol..

[15] Murakami S., Shimizu R., Romeo P.H., Yamamoto M., Motohashi H. (2014). NRF2 activation impairs quiescence and bone marrow reconstitution capacity of hematopoietic stem cells. Genes Cells.

[16] de Zeeuw D., Akizawa T., Audhya P., Bakris G.L., Chin M., Christ-Schmidt H., BEACON Trial Investigators (2013). Bardoxolone methyl in type 2 diabetes and stage 4 chronic kidney disease. N. Engl. J. Med..

[17] Senger D.R., Li D., Jaminet S.C., Cao S. (2016). Activation of the Nrf2 cell defense pathway by ancient foods: disease prevention by important molecules and microbes lost from the modern western diet. PLoS One.

[18] Sumi D., Numasawa Y., Endo A., Iwamoto N., Kumagai Y. (2009). Catechol estrogens mediated activation of Nrf2 through covalent modification of its quinone metabolite to Keap1. J. Toxicol. Sci..

[19] Heikkila R.E., Cabbat F.S. (1978). The stimulation of 6-hydroxydopamine autoxidation by bivalent copper: potential importance in the neurotoxic process. Life Sci..

[20] Pedersen J.Z., el-Sherbini S., Finazzi-Agrò A., Rotilio G. (1992). A substrate-cofactor free radical intermediate in the reaction mechanism of copper amine Oxidase. Biochemistry.

[21] Rinaldi A.C., Porcu M.C., Curreli N., Rescigno A., Finazzi-Agró A., Pedersen J.Z. (1995). Autoxidation of 4-methylcatechol: a model for the study of the biosynthesis of copper amine oxidases quinonoid cofactor. Biochem. Biophys. Res. Commun..

[22] Mandal S., Lee Y., Purdy M.M., Sayre L.M. (2000). Chemical simulation of biogenesis of the 2,4,5-trihydroxyphenylalanine quinone cofactor of copper amine oxidases: mechanistic distinctions point toward a unique role of the active site in the o-quinone water addition step. J. Am. Chem. Soc..

[23] Novoa I., Zeng H., Harding H.P., Ron D. (2001). Feedback inhibition of the unfolded protein response by GADD34-mediated dephosphorylation of eIF2alpha. J. Cell Biol..

[24] Oyadomari S., Harding H.P., Zhang Y., Oyadomari M., Ron D. (2008). Dephosphorylation of translation initiation factor 2alpha enhances glucose tolerance and attenuates hepatosteatosis in mice. Cell Metab.

[25] Lou J.J., Chua Y.L., Chew E.H., Gao J., Bushell M., Hagen T. (2010). Inhibition of hypoxia-inducible factor-1alpha (HIF-1alpha) protein synthesis by DNA damage inducing agents. PLoS One.

[26] Arias-Mayenco I., González-Rodríguez P., Torres-Torrelo H., Gao L., Fernández-Agüera M.C., Bonilla-Henao V. (2018). Acute O2 sensing: role of coenzyme QH2/Q ratio and mitochondrial ROS compartmentalization. Cell Metab..

[27] Cabello-Rivera D., Ortega-Sáenz P., Gao L., Muñoz-Cabello A.M., Bonilla-Henao V., Schumacker P.T. (2022). Oxygen regulation of breathing is abolished in mitochondrial complex III-deficient arterial chemoreceptors. Proc. Natl. Acad. Sci. U.S.A.

[28] Titov D.V., Cracan V., Goodman R.P., Peng J., Grabarek Z., Mootha V.K. (2016). Complementation of mitochondrial electron transport chain by manipulation of the NAD+/NADH ratio. Science.

[29] Tribble D.L., Jones D.P. (1990). Oxygen dependence of oxidative stress. Rate of NADPH supply for maintaining the GSH pool during hypoxia. Biochem. Pharmacol..

[30] Robledinos-Antón N., Fernández-Ginés R., Manda G., Cuadrado A. (2019). Activators and inhibitors of NRF2: a review of their potential for clinical development. Oxid. Med. Cell. Longev..

[31] Perry R.J., Kim T., Zhang X.M., Lee H.Y., Pesta D., Popov V.B. (2013). Reversal of hypertriglyceridemia, fatty liver disease, and insulin resistance by a liver-targeted mitochondrial uncoupler. Cell Metab..

[32] Fetherolf M.M., Levy-Booth D.J., Navas L.E., Liu J., Grigg J.C., Wilson A. (2020). Characterization of alkylguaiacol-degrading cytochromes P450 for the biocatalytic valorization of lignin. Proc. Natl. Acad. Sci. U.S.A.

[33] He Y., Jiang J., He B., Shi Z. (2021). Chemical activators of the Nrf2 signaling pathway in nonalcoholic fatty liver disease. Nat. Prod. Comm..

[34] Seedorf K., Weber C., Vinson C., Berger S., Vuillard L.M., Kiss A. (2022). Selective disruption of NRF2-KEAP1 interaction leads to NASH resolution and reduction of liver fibrosis in mice. JHEP Rep..

[35] Zhang J., Ouyang H., Gu X., Dong S., Lu B., Huang Z. (2024). Caffeic acid ameliorates metabolic dysfunction-associated steatotic liver disease via alleviating oxidative damage and lipid accumulation in hepatocytes through activating Nrf2 via targeting Keap1. Free Radic. Biol. Med..

[36] Fernández-Ginés R., Encinar J.A., Escoll M., Carnicero-Senabre D., Jiménez-Villegas J., García-Yagüe Á.J. (2024). Specific targeting of the NRF2/β-TrCP axis promotes beneficial effects in NASH. Redox Biol..

[37] Gu L., Du Y., Chen J., Hasan M.N., Clayton Y.D., Matye D.J. (2024). Cullin 3 RING E3 ligase inactivation causes NRF2- dependent NADH reductive stress, hepatic lipodystrophy, and systemic insulin resistance. Proc. Natl. Acad. Sci. U.S.A.

[38] Rushmore T.H., Morton M.R., Pickett C.B. (1991). The antioxidant responsive element: activation by oxidative stress and identification of the DNA consensus sequence required for functional activity. J. Biol. Chem..

[39] Gagoski D., Polinkovsky M.E., Mureev S., Kunert A., Johnston W., Gambin Y. (2015). Performance benchmarking of four cell-free protein expression systems. Biotechnol. Bioeng..

[40] Degasperi A., Birtwistle M.R., Volinsky N., Rauch J., Kolch W., Kholodenko B.N. (2014). Evaluating strategies to normalise biological replicates of western blot data. PLoS One.

[41] Nieuwenhuis S., Forstmann B.U., Wagenmakers E.J. (2011). Erroneous analyses of interactions in neuroscience: a problem of significance. Nat. Neurosci..

