# Supplementary material for: A strategy for liver selective NRF2 induction via cytochrome P450-activated prodrugs with low activity in hypoxia
Source: J Biol Chem. 2025 Apr 8;301(6):108487. doi: 10.1016/j.jbc.2025.108487 (PMC12145549; doi:10.1016/j.jbc.2025.108487)
Supplement: Figures S1–S4 [file mmc1.pdf]

Supplementary Figure 1

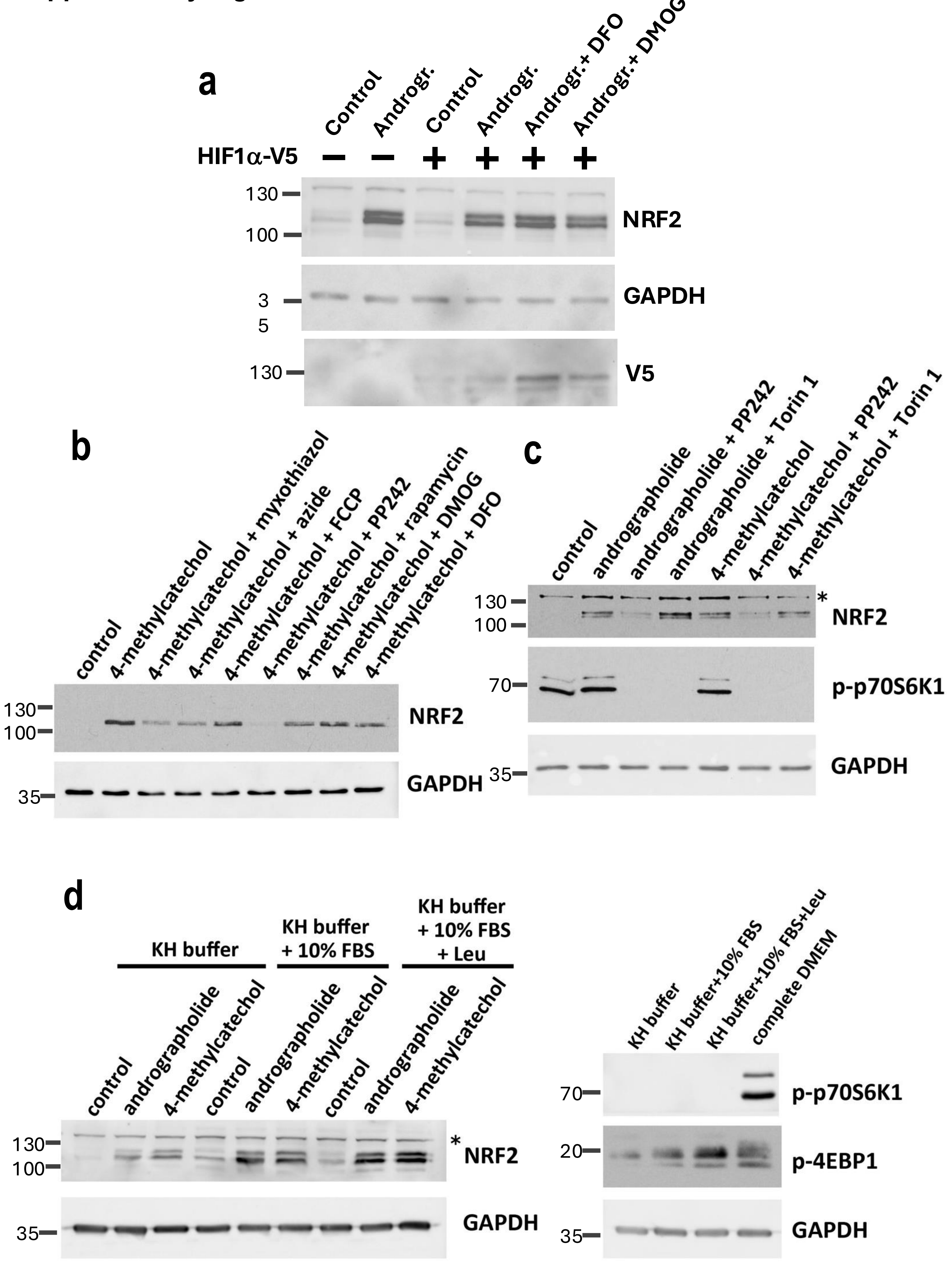

Fig.S1 The inhibition of drug induced NRF2 accumulation in hypoxia is not due to hypoxia dependent induction of HIF-1α or inhibition of mTORC1. (a) HEK293T cells were transfected with a wild type HIF-1α expression plasmid for 2 days and then incubated with 10 μM andrographolide, 200 μM desferrioxamine and 2 mM dimethyloxalylglycine (DMOG), as indicated, for 5 h, followed by cell lysis and Western blotting with the indicated antibodies. (b) HEK293T cells were incubated with 10 μM 4-methylcatechol, 1 μM myxothiazol, 2 mM sodium azide, 1 μM FCCP, 2 μM PP242, 40 nM rapamycin, 200 μM desferoxamine and 2 mM dimethyloxalylglycine (DMOG), as indicated, for 4 h, followed by cell lysis and Western blotting with the indicated antibodies. (c) To determine if the effect of PP242 is cell type dependent, we treated HeLa cells with 10 μM andrographolide, 10 μM 4-MC, 2 μM PP242, and 100 nM Torin-1 for four hours, followed by Western blotting with the indicated antibodies. (d) The cell culture media of HEK293T cells was changed to Krebs-Henseleit (KH) buffer with or without 10% fetal bovine serum (FBS) and 5 mM L-leucine, as indicated, immediately prior to the 4 h drug treatment. The cells were lysed and cell lysates used for Western blotting using the indicated antibodies. The cell lysates used in the right panel correspond to the control samples on the left. The asterisk in the NRF2 blot in (c) and (d) corresponds to a non-specific band.

Supplementary Figure 2

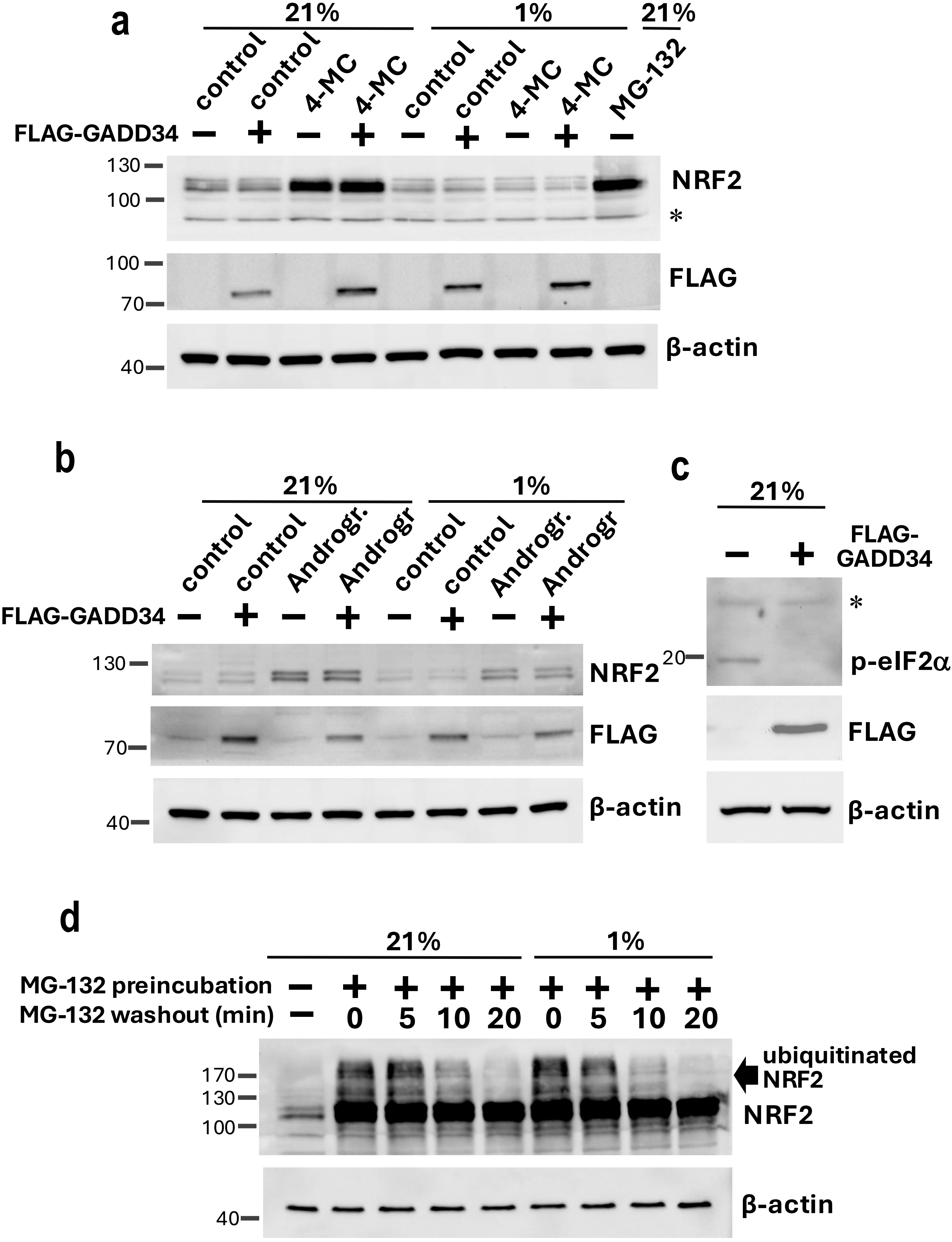

Fig.S2 The inhibition of drug induced NRF2 accumulation in hypoxia is not due to hypoxia dependent induction of eIF2α phosphorylation. (a-c) HEK293T cells were transfected for 2 days with FLAG-GADD34(amino acids 263 to 674) (Lou, J. J., Chua, Y. L., Chew, E. H., Gao, J., Bushell, M., and Hagen, T. (2010) Inhibition of hypoxia-inducible factor-1alpha (HIF-1alpha) protein synthesis by DNA damage inducing agents. *PLoS One* **5**, e10522), as indicated, in order to inhibit eIF2α phosphorylation. The C-terminal GADD34 fragment is sufficient to recruit PP1 to eIF2α and cause its dephosphorylation (Novoa, I., Zeng, H., Harding, H. P., and Ron, D. (2001) Feedback inhibition of the unfolded protein response by GADD34-mediated dephosphorylation of eIF2alpha. *J. Cell Biol.* **153**, 1011–1022; Oyadomari, S., Harding, H. P., Zhang, Y., Oyadomari, M., and Ron, D. (2008) Dephosphorylation of translation initiation factor 2alpha enhances glucose tolerance and attenuates hepatosteatosis in mice. *Cell Metab.* **7**, 520–532). The cells were treated with 10 μM 4-MC, 10 μM andrographolide or 10 μM MG-132, as indicated, for 4 h at 21% or 1%. Prior to drug addition to the hypoxic cell cultures, the cells were preequilibrated at 1% oxygen for 30 min. After the 4 h incubation time, the cells were lysed and the cell lysates subjected to Western blotting using the indicated antibodies. (d) To determine the time course of the degradation of polyubiquitinated NRF2 at 21% versus 1%, HEK293T cells were treated for 4 h with the proteasome inhibitor MG-132 (20 μM). MG-132 was then washed out and replaced with media containing the Cullin E3 ligase inhibitor MLN4924 (2 μM) to inhibit new NRF2 polyubiquitination. The degradation of polyubiquitinated NRF2 was assayed after the indicated time periods of incubating the cells at 21% or 1% oxygen by lysing the cells and blotting the cell lysates with NRF2 antibody. For the 1% oxygen condition, the cells were placed into the hypoxic incubator 45 min before the MG-132 washout. The fresh media containing MLN-4924 was also preequilibrated for 45 min at 1% oxygen. The asterisk in (a) and (c) corresponds to a non-specific band.

Supplementary Figure 3

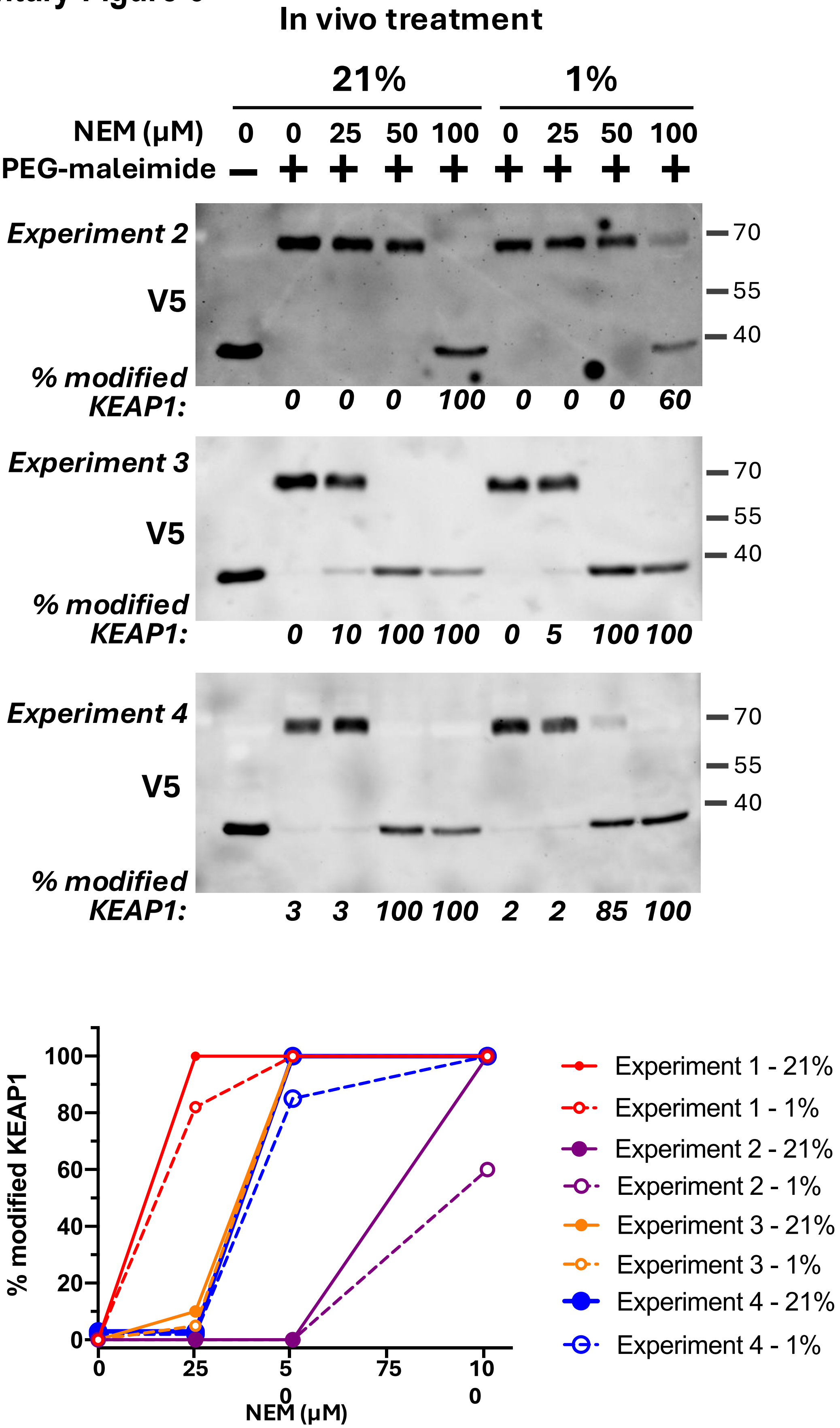

Fig.S3 Hypoxia inhibits drug induced cysteine adduct formation in KEAP1 in intact cells. The experiments were performed as described in Fig.12a.

Supplementary Figure 4

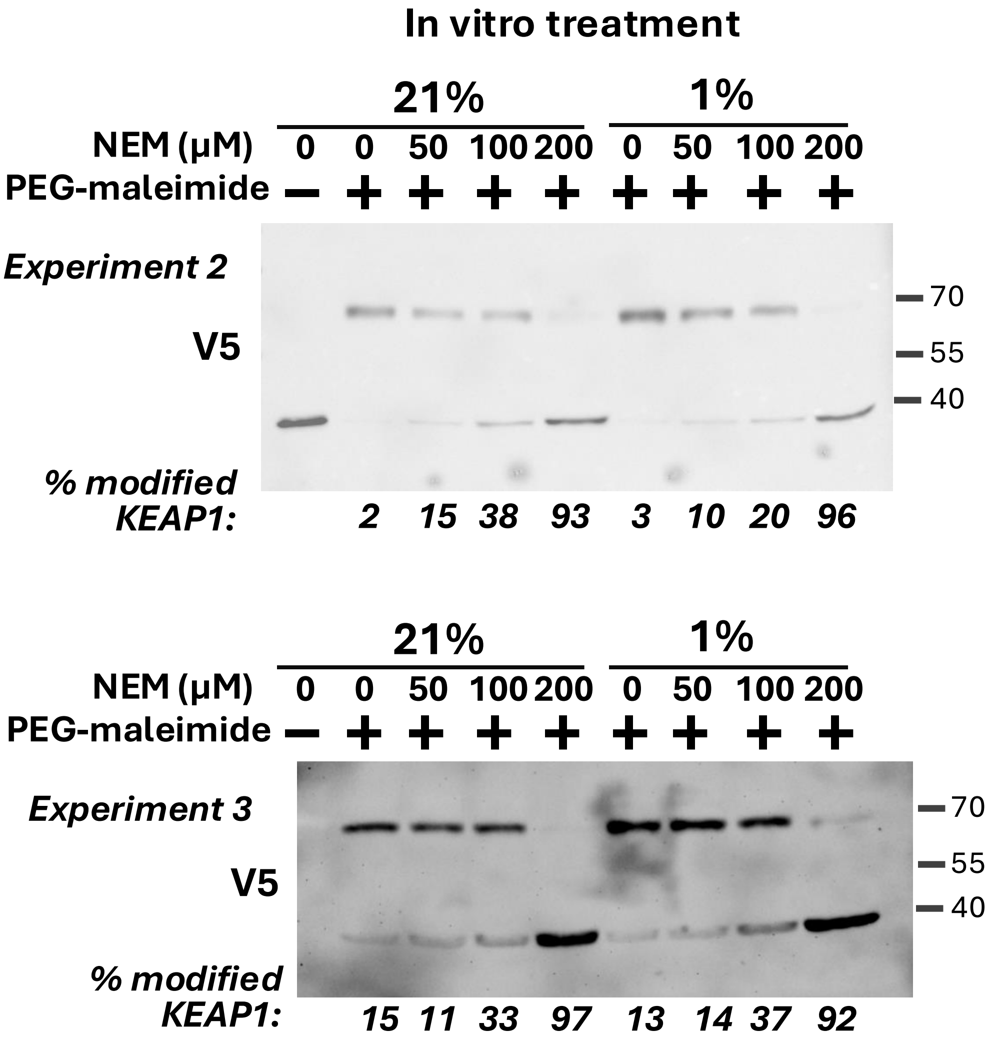

Fig.S4 Hypoxia does not affect drug induced cysteine adduct formation in KEAP1 *in vitro*. The experiments were performed as described in Fig.12b. In the bottom panel, the (-)PEG maleimide control is not included.
